# Venetoclax in Relapsed/Refractory Acute Myeloid Leukemia: Are Supporting Evidences Enough?

**DOI:** 10.3390/cancers14010022

**Published:** 2021-12-21

**Authors:** Serena Brancati, Lucia Gozzo, Giovanni Luca Romano, Calogero Vetro, Ilaria Dulcamare, Cinzia Maugeri, Marina Parisi, Laura Longo, Daniela Cristina Vitale, Francesco Di Raimondo, Filippo Drago

**Affiliations:** 1 Clinical Pharmacology Unit, Regional Pharmacovigilance Centre, University Hospital of Catania, 95123 Catania, Italy; serena.brancati@gmail.com (S.B.); longolaura@hotmail.com (L.L.); danielac.vitale@gmail.com (D.C.V.); f.drago@unict.it (F.D.); 2 Department of Biomedical and Biotechnological Sciences, University of Catania, 95123 Catania, Italy; giovanniluca.romano@unict.it; 3 Haematology Unit, A.O.U. Policlinico “G. Rodolico-S. Marco”, 95123 Catania, Italy; gerovetro@gmail.com (C.V.); ilarialaruna@gmail.com (I.D.); maugericinzia@hotmail.com (C.M.); marinaparisi@hotmail.it (M.P.); diraimon@unict.it (F.D.R.); 4 Department of Chirurgia Generale e Specialità Medico-Chirurgiche, University of Catania, 95123 Catania, Italy; 5 Centre for Research and Consultancy in HTA and Drug Regulatory Affairs (CERD), University of Catania, 95123 Catania, Italy

**Keywords:** venetoclax, acute myeloid leukemia, relapsed/refractory, off-label, regulatory issue

## Abstract

**Simple Summary:**

Venetoclax (VEN) is a potent oral inhibitor of the anti-apoptotic molecule BCL2, approved for adults with chronic lymphocytic leukemia (CLL), and recently for naïve acute myeloid leukemia (AML) unfit for intensive chemotherapy. Therefore, VEN is not approved for relapsed/refractory (R/R) AML patients; consequently, this use falls within the so-called off-label use. This review provides evidence of the role of VEN-based therapy in R/R AML patients, including data from clinical trials and from retrospective studies.

**Abstract:**

Despite the progress in the development of new therapeutic strategies, relapsed/refractory (R/R) acute myeloid leukemia (AML) still represents a high unmet medical need. Treatment options in this setting include enrollment into clinical trials, allogeneic stem cell transplantation and/or targeted therapy. Nevertheless, it is associated with poor outcomes. Thus, the development of new treatments, which could ameliorate the prognosis of these patients with a good safety profile are highly demanded. Recently, venetoclax (VEN) has been approved for naïve AML patients unfit for intensive chemotherapy. In this regard, regimens including VEN could represent a valuable treatment option even in those with R/R disease and several studies have been conducted to demonstrate its role in this clinical setting. This review aims to summarize the current evidence on the use of VEN regimens in the treatment of R/R AML.

## 1. Introduction

Venetoclax (VEN) is a potent oral inhibitor of the anti-apoptotic molecule BCL2, used to treat adults with chronic lymphocytic leukemia (CLL), in association with obinutuzumab in patients who have not previously been treated or with rituximab in patients who have received at least one previous treatment [1].

It is also used as monotherapy in patients with 17p deletion or *TP53* mutation who cannot be treated with or have failed a B-cell receptor pathway inhibitor or in the absence of these genetic changes in adult patients who have failed both chemo-immunotherapy and a B-cell receptor pathway inhibitor [1].

In the last years, this drug demonstrated to be safe and effective in patients with other hematological diseases, in particular acute myeloid leukemia (AML). The median age of AML patients at diagnosis is 68 years and this population has often limited effective treatment options due to ineligibility for intensive chemotherapy [2,3]. Low-dose cytarabine (LDAC) or hypomethylating agents (azacitidine, decitabine) can be used in this population but have been associated with poor response (complete remission, CR, plus CR with incomplete blood count recovery, CRi, rates less than 30% and median survival <6 months) [4,5,6]. Preliminary results and subsequent confirmatory data showed that the association of VEN + hypomethylants/LDAC was associated with higher response rate and better overall survival [7,8,9].

Therefore, an extension of indication has been granted for VEN by the Food and Drug Administration (FDA) for the use in combination with azacitidine or decitabine or LDAC for the treatment of newly diagnosed AML who are unfit for intensive chemotherapy due to age or comorbidities [10,11]. Moreover, this use was approved in 2021 in Europe, only in combination with a hypomethylating agent (HMA) for the treatment of adult patients with newly diagnosed AML ineligible for intensive chemotherapy [12].

However, despite the progress in the development of new therapeutic strategies, relapsed/refractory (R/R) disease is associated with poor outcomes and still represents an unmet medical need [13,14,15]. Treatment options for these patients include enrollment into clinical trials, allogeneic stem cell transplantation (the only potential curative option), targeted therapy (such as gilteritinib for AML with an *FLT3* mutation).

Thus, the development of new treatment modalities, which could ameliorate the prognosis of these patients with a good safety profile, is highly demanded.

In this regard, regimens including VEN could represent a valuable treatment option even in younger patients with R/R AML and in recent years several studies have been conducted to demonstrate its role in this clinical setting [15].

This review aims to analyze the current evidence on the use of VEN-containing regimens in the treatment of R/R AML.

## 2. Mechanism of Action and Resistance in AML

The BCL-2 family consists of various pro-apoptotic and anti-apoptotic molecules, which regulate the intrinsic apoptotic pathway and have been implicated in the cell survival but also tumorigenesis in many hemato-oncological malignancies [16]. This pathway is activated in response to stress or DNA damage, and leads to the formation of pores in the outer mitochondrial membrane through effector proteins (BAX and BAK), resulting in the release of cytochrome C into the cytosol, caspase-9 activation, and proteolytic cell death.

The overexpression of BCL-2 has been associated with cell survival and apoptosis escape, but also with therapy resistance [17].

Pre-clinical studies have shown that stem cells depend on BCL-2 for survival, and inhibition of this molecule can lead to apoptosis and eradication of these cells [18,19,20,21,22,23]. In particular, aberrant overexpression of BCL-2, together with other anti-apoptotic proteins as BCL-XL and MCL-1, has been detected in AML cells [24,25,26], where suppressed mitochondrial-modulated programmed cell death, supports cell survival [27], mediates chemoresistance and confers survival benefits [28].

In preclinical studies, the oral BH3 mimetic highly selective for BCL-2, VEN, exhibited potent anti-leukemic activity in AML cell lines, xenograft murine models and patient samples [18]. The drug acts by binding BCL-2 causing the release of sequestered pro-apoptotic signaling proteins BAX and BAK [29,30]. Alternatively, it might also lead to cell death by destabilizing the proton gradient across the mitochondrial inner membrane [31]. Moreover, the combination of VEN + azacitidine inhibits amino acid metabolism, which has been recognized as fundamental to leukemia stem cell survival [32]. This inhibition reduces oxidative phosphorylation and induces cell death, in particular in de novo patients [19,33]. In the R/R setting, an upregulation of fatty acid metabolism has been recognized as a potential compensatory metabolic pathway; therefore, the change in metabolic phenotype of relapsed disease may be responsible for lower VEN efficacy [34,35].

Whatever the mechanism of action, the ultimate effect is the mitochondrial outer membrane permeabilization (MOMP), release of cytochrome C to the cytoplasm and decreased ATP production, formation of cytosolic apoptosome complexes, and subsequent caspase activation and apoptosis [36,37]. Notably, as also reported in multiple myeloma and CLL [38,39], VEN sensitivity is strongly and inversely correlated with the BCL-2/MCL-1 ratio, with a loss of AML cell sensitivity when high levels of MCL-1 are expressed [18,40,41,42,43].

Indeed, overexpression of MCL1 plays a major role in the pathogenesis of leukemia and mediates VEN resistance if BCL2 is not the primary anti-apoptotic driver. In this case, concurrent inhibition of MCL1 can overcome VEN resistance [20,44,45,46,47,48,49,50].

Previous studies showed an improvement of efficacy of VEN in combination with drugs that downregulated MCL1 and/or BCL-XL, such as HMAs and cytarabine which exert a synergistic effect with VEN to interfere with the energy metabolism and kill AML tumor cells [20,22,23,51,52,53,54,55,56,57].

It is noteworthy that patients with *IDH1/2*-mutations treated with VEN combinations showed higher and durable responses, and longer survival compared to other subgroups. Indeed, *IDH1/2* mutations induce BCL-2 dependence, making AML cells particularly sensitive to VEN, as a single agent or in combination with other agents [7,58,59,60,61].

On the contrary, *FLT3-ITD* mutation may produce primary resistance to VEN by enhancing the expression of other anti-apoptotic molecules such as BCL-XL and MCL-1 [47,61,62] and responses to VEN-based combinations have been lower and short-lived [7,58,59]. The combination of VEN with FLT3-tyrosine kinase inhibitor (TKI) induced more durable tumor regression in FLT3-mutant AML cell lines [62].

## 3. Clinical evidence in R/R AML

### 3.1. Clinical Trials

Currently, only few clinical trials (phase I/II) with VEN-based regimens have been performed in R/R AML patients (Table 1).

A phase II study showed only a modest activity (CR/CRi = 19%) of VEN monotherapy administered in R/R AML patients [46].

Given these findings and the good results of VEN-combination regimens in the front-line setting, a single-center phase II trial evaluated safety and efficacy of VEN in combination with 10-days decitabine in a large cohort (*n* = 168) of patients, including those with treated secondary AML (*n* = 28), or R/R AML (*n* = 55) [63]. Patients were treated with intravenous decitabine (20 mg/m^2^) for 10 days + oral VEN (400 mg) daily for induction, followed by decitabine for 5 days with daily VEN for consolidation. Primary endpoint was overall response rate (ORR) [complete response (CR) + CR with incomplete blood count recovery (CRi) + partial response (PR) + morphological leukemia free state (MLFS)] according to the revised IWG criteria [94]. Secondary endpoints included duration of response, overall survival (OS), safety, and the proportion of patients who transitioned towards hematopoietic stem-cell transplantation (HSCT). R/R patients had a median age of 62 years (43–73) and received a median of 2 (1–3) previous therapies, including HMA (*n* = 25, 45%), intensive chemotherapy (*n* = 42, 76%), HMA and intensive chemotherapy (*n* = 12, 22%), and SCT (*n* = 18, 33%). Overall, the ORR was 74% (*n* = 125, 95% CI: 67–80), with CR/CRi in 103 (61%) patients (95% CI: 54–68); ORR in patients with R/R AML was 62% (*n* = 34/55, 95% CI: 49–74), with CR in 13 patients (24%), CRi in 10 patients (18%), and MLFS in 10 patients (18%). Minimal residual disease (MRD) negativity was globally attained in 60 (58%, 95% CI: 49–67) of 103 responding patients [14 of 26 (54%) patients with R/R AML].

The median OS was 7.8 months (95% CI: 5.4–13.3) in R/R AML patients, 18.1 months (95% CI: 10–not reached) in newly diagnosed AML, 7.8 months (95% CI: 2.9–10.7) in patients with untreated secondary AML, and 6.0 months (95% CI: 3.4–13.7) in patients with treated secondary AML. Median duration of response (CR or CRi) was not reached (95% CI: 9.0–not reached) in newly diagnosed AML, 5.1 months (95% CI: 0.9–not reached) in untreated secondary AML, not reached (95% CI: 2.5–not reached) in previously treated secondary AML, and 16.8 months (95% CI: 6.6–not reached) in R/R AML patients [9.8 months (95% CI: 3.2–not reached) in patients with four or more previous cycles of HMAs and 12.9 months (95% CI: 6.6–not reached) in those with previous intensive chemotherapy]. Patients in the R/R group who had particularly favorable outcomes included patients with diploid cytogenetics, *NPM1*, *IDH1/IDH2*, and *FLT3* mutations.

Exploratory subgroup analyses among 83 previously treated patients (also including R/R patients) showed a CR or CRi rate of 37% (95% CI: 22–55) in those having received at least four previous cycles of HMAs, of 36% (24–51) in those with previous intensive chemotherapy, and of 27% (14–46) in those with previous SCT. The CR or CRi rate was 48% (95% CI 30–67) for patients receiving first salvage therapy for AML and 40% (20–64) for those receiving second salvage therapy. Twenty-three patients underwent SCT after a median of three cycles of treatment [interquartile range (IQR) 2–3]. Responding patients transitioning to SCT had the most durable responses with median OS not reached (95% CI: 13.0–not reached) for treatment-naïve patients and 22.1 months (95% CI: 6.8–not reached) in previously treated groups.

Globally, 261 treatment-emergent adverse events (TEAEs) in 134 patients were reported, with 193 grade 3 or 4, the most common of which were infections with prolonged grade 3 or 4 neutropenia (*n* = 79, 47% patients)—that forced most patients to reduce the duration of VEN in subsequent cycle—and febrile neutropenia (*n* = 49, 29% patients), and six grade 5 AEs, related to neutropenic infections. The 30-day mortality for all patients was 3.6% (*n* = 6, 95% CI 1.7–7.8), and the 60-day mortality was 10.7% (*n* = 18, 6.9–16.9).

In summary, VEN with 10-day decitabine achieved an excellent response both in untreated AML patients and in those previously treated (including R/R AML), and showed an acceptable tolerability, with expected TEAEs (infections with grades 3 or 4 neutropenia and febrile neutropenia) and a low 30-day and 60-day mortality.

Recently, the results of a phase Ib/II study evaluating the combination of fludarabine, cytarabine, granulocyte colony-stimulating factor, and idarubicin with VEN in naïve and R/R-AML have been published [64]. Sixty-eight patients have been enrolled, including 39 with R/R disease, with a median age of 46 years (range, 20–73). The ORR was 75% in those enrolled in the phase Ib portion (composite CR = 75%), and 70% in the phase IIb (composite CR = 61%). MRD-negativity was attained in 69% of R/R patients. Median OS was not reached after 12 months. Forty-six percent of R/R-AML patients proceeded to allogeneic HSCT, with a significant improvement in OS (median OS, NR; 1-year OS, 87%).

Therefore, this regimen represents an effective treatment even in R/R-AML patients, associated with high rate of remission and the possibility to be a successful bridge to transplantation.

Several clinical trials with regimen including VEN in R/R AML patients are still ongoing. Table S1 shows the details of these studies, in particular the trial design, the number of patients to enroll, the study interventions, the primary outcome measures, and the estimated completion date.

### 3.2. Observational and Real-World Studies

Since FDA approval in 2018 for upfront use in AML, VEN-combination regimens have been widely used in real-world settings as salvage regimens, with several published retrospective reports documenting activity in patients with R/R AML (Table 1).

A first report of 43 R/R myeloid patients [39 (91%) with AML, 2 with MDS, and 2 with blastic plasmacytoid dendritic cell neoplasm]—the majority of which were over age 65 (58%) and ≥2 salvage-treatment (*n* = 36, 84%), including prior HMA in 77% and prior allogenic SCT in 12%—who received VEN-based salvage therapy, most commonly in combination with HMAs, documented an ORR of 21% (*n* = 9), with 2 (5%) CR, 3 (7%) CRi and 4 (9%) MLFS [65]. All 9 patients with ORR responded within the first cycle and 2 successfully transitioned to allogenic SCT. The median OS was 3.0 months overall and 4.8 months in the 9 responders (range 0.5–8.0) and the estimated 6-month survival was 24%. Treatment was discontinued in 38 patients due to the lack of response or disease progression (*n* = 29, 77%), death (*n* = 7, 16%), or transition to allogeneic SCT (*n* = 2, 5%). In regard to molecular/cytogenetic risk, 3/11 (27%) patients with *IDH* mutations, 4/8 (50%) *RUNX1*-mutated patients and 2/10 (20%) *TP53*-mutated patients (with a concurrent *RUNX1* mutation) achieved an objective response. The most common complications in this heavily pretreated, older, and high-risk R/R population were grade ≥ 3 neutropenia and grade ≥ 3 infections, mainly pneumonia (*n* = 17, 40%). Several retrospective studies analyzed the therapeutic response according to the specific molecular subgroup. Sub-analysis of specific molecular mutations in the retrospective study by Aldoss et al. [66] showed a response rate of 67% for *IDH 1/2* mutations (similarly to previous reports [7,8,46]), 44% for *FLT3* mutations, and 67% for *TP53* mutations, suggesting a good clinical activity for the combination of VEN + HMAs even in patients who would be expected to respond poorly to conventional chemotherapy. These results are in line with the single-center retrospective analysis of a cohort of 90 adults with R/R AML treated with at least one cycle of VEN + HMA [67]. In this study, different conventional predictors of poor response (as failure of prior HMA therapy or prior allogenic SCT) were not associated with response rate. Moreover, high-risk mutations such as *TP53* and *FLT3* were not associated with a lower rate of response compared to other mutations. Only *TP53* mutation was associated with reduced LFS in univariate analysis (*p* = 0.01), but not in multivariate analysis (*p* = 0.074).

A retrospective analysis of 48 adult patients with R/R AML treated with VEN + azacitidine showed the highest response (75%) in patients with *IDH1/2* mutations (*n* = 12) and with *RUNX1* mutation (*n* = 4) [82]. This trend of a better response for *IDH1/2* mutations was also confirmed in multivariate analysis (OR = 4.54, 95% CI: 0.911–22.617, *p* = 0.07). In contrast, the response was independent of classic adverse factors as *FLT3-ITD*, *RUNX1* or *TP53* mutations. The median OS for the whole population was 9.6 months, significantly longer in those with *IDH1/2* mutations (not reached vs. 3.2 months in wild-type, *p* < 0.001; HR = 0.069, 95% CI: 0.006–0.726, *p* = 0.026), and significantly shorter in case of *TP53* mutation (4.7 months vs. not reached in wild-type, *p* < 0.001; HR = 22.855, 95% CI: 2.549–204.949, *p* = 0.005).

The results of a small retrospective study on 12 patients with R/R AML carrying the *NPM1* mutation (5 with molecular persistence and 7 with progression or relapsed), showed a high rate of CR with MRD negativity (92%) after 1–2 cycles of VEN + LDAC or azacitidine [86]. Median relapse-free survival was not reached in the 5 responders with previous molecular persistence (median follow-up = 20 months), and none of them experienced disease progression or received an allogenic HSCT for consolidation.

Another retrospective study by Aldoss et al. [68], including 32 adult patients all with *TP53*-mutated AML (16 R/R and 15 newly diagnosed) treated with VEN + HMA, reported a global response rate of 52% (*n* = 16), with 7 patients experiencing CR and 9 CRi (38% CR/CRi rate in R/R). This represents a good result considering the poor outcomes reported in *TP53*-mutated AML patients treated with conventional combination chemotherapy [95]. Interestingly, patients with more than one *TP53* mutation obtained a higher CR/CRi rate (78% vs. 41%, *p* = 0.062). Another study by Aldoss et al. [69] revealed a response of 42% among R/R AML patients with *FLT3* mutations, better in those who were naïve for *FLT3*-based TKIs (37% for patients with prior exposure vs. 50% in naïve).

The potential role of mutations as predictive factors for response to VEN-based therapy has been assessed in a cohort of 40 R/R AML patients [70]. *NPM1*, *RUNX1*, or *SRSF2* mutations have been associated with higher CR/CRi rates and *RUNX1* with longer OS. On the contrary, *FLT3-ITD*, *TP53*, or *DNMT3A* mutations resulted into worse outcome.

Piccini et al. recently showed a particularly favorable outcome for HSCT-naïve patients and aged >60 years, even in the multivariate analysis [71]. Median OS was 10.7 months, longer in the group with *NPM1* mutation (median not reached) and shorter in the group with both *NPM1-FLT3/ITD* mutations (2.3 months).

Regarding the use of VEN as salvage therapy post allogenic HSCT, in a cohort of 21 post-transplant relapsed AML patients who received off-label VEN-based regimens (mainly with HMAs), the observed response rate was 42.1% (*n* = 8, 5 CR and 3 CRi) [73]. None of the four patients with complex karyotype and *TP53* mutations responded to therapy. The median OS was 7.8 months (range 0.2–12.1 months), with a significantly longer OS in patients achieving CR/CRi (*p* = 0.005). Only one patient with CR/CRi progressed prior to the data cut-off, after 9.2 months on therapy. Another retrospective analysis conducted on 29 patients with post allo-HCT AML relapse treated with VEN regimens as salvage therapy [76] showed a 38% response rate, including CR/CRi in 8 subjects. The median duration of response was 7 months (1–11) and median OS was 79 days (2–403), better in responders (403 vs. 55 days, *p* < 0.0001). A high response rate was 61.5% (including 26.9% of CRi) which has been observed even by Zhao et al. [77] in 26 patients with AML relapsed after allo-HSCT treated with VEN + azacitidine and donor lymphocyte infusion. In the 6 patients relapsed after allogeneic HCT and retrospectively observed by Ram et al. [85], CR/CRi was achieved in 67% of the patients (*n* = 4) and the median OS was 12.4 months.

On the contrary, no CR was reported in patients who received a prior allogenic HSCT in a single-center analysis of 21 patients with a R/R myeloid malignancy (*n* = 20 AML and *n* = 1 MDS) treated with the combination of VEN + HMA (*n* = 8) or VEN + LDAC (*n* = 16), reported an ORR of 28.6% (95% CI: 11.3–52.2%) (*n* = 6). Zucenka et al. [75] compared the outcome of R/R patients after alloSCT treated with VEN + LDAC + actinomycin D (*n* = 20) with those receiving fludarabine + cytarabine + idarubicin (FLAG-Ida; *n* = 29). Patients treated with the VEN regimen obtained better results compared to the FLAG-Ida group (ORR 75% vs. 66%; *p* = 0.542; CR/CRi rate 70% vs. 34%; *p* = 0.02; OS 13.1 vs. 5.1 months; *p* = 0.032).

It is noteworthy that in this setting of heavily pretreated patients responses were obtained even with low-dose VEN (100 mg/day) + decitabine (20 mg/m^2^ day 1–5) (*n* = 8) or azacitidine (75 mg/m^2^ for 7 days), with minimal toxicities [74].

Several responders successfully proceeded to allogenic HSCT [13].

In this regard, Zappasodi et al. [84] specifically explored the utility of VEN with azacitidine as a bridge to allogeneic SCT in 10 heavily pretreated patients with refractory AML. ORR was 60% (*n* = 6), with 4 CR, 1 CRi, and 1 MLFS. HSCT was performed in all 6 responders and in 1 non-responder with a consistent reduction of blasts in the bone marrow and resolution of the thrombocytopenia. Five of the transplanted patients were alive at the time of the analysis and four were in CR. Median OS was 8.9 months (range 2–19) and 11.7 months among transplanted patients.

The analysis of Stahl et al. [87] in a large cohort of real-world patients (*n* = 86) with R/R AML, reported a significantly higher response rate in patients treated with VEN + azacitidine or decitabine than in those treated with VEN + LDAC (49% vs. 15%; OR = 5.43, 95% CI: 1.55–19, *p* = 0.008; 25% vs. 15%; OR = 1.92, 95% CI: 0.44–8.31, *p* = 0.38). Moreover, the median OS was significantly improved in responders (HR = 0.1, 95% CI: 0.04–0.3, *p* = 0.001) and in those treated with VEN + azacitidine (25 months), compared to VEN + decitabine (5.4 months) (*p* = 0.13) and VEN + LDAC (3.9 months) groups (*p* = 0.003). Patients with *NPM1* mutations reached a higher response rate, whereas in those with adverse cytogenetics and mutations in *TP53*, *KRAS/NRAS* and *SF3B1* a decreased OS was observed.

Sixty-five R/R patients treated with 10-day decitabine and VEN were compared to 130 patients treated receiving intensive chemotherapy [89]. Patients in the VEN group obtained a significantly higher response (ORR 60% vs. 36%; odds ratio [OR], 3.28; *p* < 0.001), including higher CRi (19% vs. 6%; OR, 3.56; *p* = 0.012), MRD negativity (28% vs. 13%; OR, 2.48; *p* = 0.017), and longer median event-free survival (5.7 vs. 1.5 months; hazard ratio [HR], 0.46; 95% CI, 0.30–0.70; *p* < 0.001), as well as median OS (6.8 vs. 4.7 months; HR, 0.56; 95% CI, 0.37–0.86; *p* = 0.008).

In regard to safety, the most common AEs were neutropenia, and febrile neutropenia, including serious events, which can lead to drug interruption and/or hospitalization. Overall, the drug had an acceptable safety profile, especially in patients with optimal blood count prior to treatment.

All these retrospective data support the role of VEN-based regimen as an effective treatment in R/R-AML patients, even in those heavily pre-treated and relapsed after HSCT. The role of predictors of good response should be confirmed in larger specific clinical trials.

## 4. The Regulatory Perspective: The Off-Label Use Regulation

Currently VEN is not approved for R/R AML patients; therefore this use falls within the so-called off-label use [96,97,98,99,100,101,102]. EMA defined off-label use as “situations where the medicinal product is intentionally used for a medical purpose not in accordance with the authorized product information” [72]. The major advantage of off-label use is the potential satisfaction of unmet medical needs; however, it could increase the risk of inappropriate use, medical error and adverse events due to the uncertainty about the drug value in the off-label indication, related to the lack of conclusive data supporting its benefit–risk ratio [103]. In this context, it is crucial and ethically acceptable that patients should always be well-informed about the proposed unauthorized use, about the available evidence and the reasons why it represents the best therapeutic option, so that they can consciously provide their consent to the off-label treatment.

This use is not regulated in Europe, but member states adopted specific regulation in some cases [72]. For example, the Italian Law 94/1998 allows physicians to perform off-label prescriptions in individual and exceptional cases, if the prescriber assumes the responsibility of the prescription, an adequate mandatory informed consent of the patient is provided, and efficacy and safety results derived from at least phase II clinical trials are available [104]. The cost of these prescriptions is not covered by the national health system (NHS). In addition, the Italian Law 648/96 includes the reimbursement of an off-label use supported by evidence from at least phase II trials and recognized by the national regulatory authority. In this case, patient associations, scientific societies, and clinical centers may request to the national competent authority the approval of new therapeutic use of effective and safe medicines beyond the interest of pharmaceutical companies. Finally, the off-label use can be covered in Italy within the “5% Fund” according to Law 326/2003, ensuring access for rare or serious diseases [105].

In France the Recommandations Temporaires d’Utilisation (RTU) ensures nationwide access to off-label drugs according to criteria for appropriate use and monitoring defined in the light of clinical evidence [106,107], as well as the Law 648/1996 in Italy [108].

Since March 2020, the Italian NHS covers the use of VEN in combination with HMAs for adult patients with newly diagnosed AML not eligible for intensive induction chemotherapy [109,110]; on the contrary, the use in R/R is not approved and falls within the Italian Law 94/1998, by which physicians can perform off-label prescriptions (not covered by the NHS) only in individual and exceptional cases [111]. This represents, to date, a limitation due to the exceptionality and not systematicity that should characterize the prescription.

In recent years, VEN has been nominally authorized by the Agenzia Italiana del Farmaco (AIFA) for several patients [71]; however, this can be a time-consuming administrative procedure which can complicate healthcare management.

Based on current evidence including phase II trials, the use of VEN in R/R AML patients could be recognized according to Law 648/96, improving patients’ access.

## 5. Conclusions

This review provides evidence of the role of VEN-based therapy in R/R AML patients, although most data came from retrospective studies.

Globally, these findings suggest an efficacy of VEN in combinations with HMAs (decitabine and azacitidine) or LDAC not only in the upfront setting but also in R/R AML, with an acceptable safety profile, comparable to those observed in other conditions, like CLL, with persistent cytopenias, infections, and transfusion requirements as major toxicities. VEN regimens can lead to high response rates with moderately durable remission and survival and can bridge patients to transplant. The considerable variability observed regarding clinical and mutational predictors of response and survival might be explained by the heterogeneity in disease biology and prior therapies received in the context of R/R AML patients.

In summary, despite the role of VEN-based combination regimens in the treatment of R/R AML patients should be further confirmed and optimized in additional prospective controlled clinical trials (currently ongoing), these combinations may represent a good salvage option in R/R AML in particular in case clinical trials are not available, other intensive chemotherapy regimens or HSCT have already failed, or in those who cannot tolerate intensive chemotherapy due to their age or performance status.

## Figures and Tables

**Table 1:** Main efficacy and safety results from clinical trials and observational studies.

Author, Year	Study Design	Age (Range)	Number of Patients	Arms and Interventions	Efficacy	Safety
Konopleva et al., 2016 [46]	Phase II open-label, single-arm study	Median age 71 (19–84)	32 (including 30 R/R AML)	VEN 800 mg/die with a stepwise ramp-up dosing	ORR = 19% (6/32); 6% (2) = CR; 13% (4) = CRi. Median duration of VEN therapy in responders = 144.5 days (83–256); Median duration of CR = 48 days.	VEN monotherapy was generally well tolerated. Most common AEs = nausea, diarrhea, hypokalemia, vomiting, and headache. Most common grade 3/4 AEs = febrile neutropenia, hypokalemia, pneumonia, hypotension, and urinary tract infection.
DiNardo et al., 2020 [63]	Phase II study	Median age 62 (43–73) for R/R patients	168 (including 55 R/R AML)	VEN 400 mg/die + 10-days decitabine 20 mg/m^2^	ORR = 74% (*n* = 125, 95% CI: 67–80); CR/CRi = 61% (95% CI: 54–68); ORR in patients with R/R AML = 62% (*n* = 34/55, 95% CI: 49–74); CR = 24%, CRi = 18%, MLFS 18%. Median duration of response (CR or CRi) = 16.8 months (95% CI: 6.6–not reached) in R/R AML patients.	261 TEAEs in 134 patients (193 grade 3 or 4). The most common = infections with prolonged grade 3 or 4 neutropenia, and febrile neutropenia, and 6 grade 5 AEs, related to neutropenic infections.
DiNardo et al., 2021 [64]	Phase Ib/II study	Median age 46 (range, 20–73)	68 (including 39 with R/R AML)	FLA-Ida + VEN	ORR = 75% in the phase Ib portion and 70% in the phase IIb. CR = 67% of R/R-AML patients (including 57% patients with prior alloHSCT). 100% CR rate and 83% 12-month OS = molecular subgroups (NPM1, IDH1, or IDH2) conferring sensitivity to VEN-based therapy in R/R-AML.	Grade 3 and 4 AEs = ≥10% of patients included febrile neutropenia (50%), bacteremia (35%), pneumonia (28%), and sepsis (12%). Deaths in R/R patients, including 4 non-responders (2 sepsis; 1 pneumonia; 1 pulmonary hemorrhage) and 2 responders (sepsis and hemophagocytic syndrome).
DiNardo 2018 [65]	Retrospective study	Median 68 (25–83)	43 R/R myeloid patients (including 39 with AML)	VEN-based salvage therapy at the median dose of 200 mg daily (range 100–800 mg), most commonly in combination with a HMA	ORR = 21% (*n* = 9); CR = 2 (5%); CRi = 3 (7%); MLFS = 4 (9%)	The most common AEs = grade ≥ 3 neutropenia and grade ≥ 3 infections, mainly pneumonia, bloodstream infections (gram − or gram + bacteria), cellulitis, invasive fungal infections and urinary tract infections.
Aldoss et al. 2018 [66]	Retrospective study	Median 62 (19–81)	33 R/R AML	VEN 400 mg daily + decitabine (20 mg/m^2^/day) or azacitidine (75 mg/m^2^/day for 7 days per cycle, 10-days or 5-days course)	ORR = 64% (*n* = 21); CR = 30% (*n* = 10); CRi = 21% (*n* = 7); MLFS = 12% (*n* = 4)	Serious neutropenic infections = 19 during the first cycle, including a prolonged cytopenia lasting more than 6 weeks, and 17 for the subsequent cycles, complicated by serious AEs (11 sepsis, 5 pneumonia, 3 colitis and diarrhea, 2 atrial fibrillation, and 2 acute renal failure).
Aldoss et al., 2019 [67]	Single-center retrospective analysis	59 years (18–81)	90 adults with R/R AML	VEN + HMA	ORR = 46% (*n* = 41); CR = 26%; CRi = 20%. In multivariate analysis, reduced CR/CRi only with ELN genetic risk (OR: 0.25; 95% CI: 0.09–0.67); better CR/CRi = whit the *ASXL1* (OR: 4.88; 95% CI: 1.02–25.67, *p* = 0.029) or *TET2* (OR 12.21; 95% CI: 1.19–636.50, *p* = 0.023) mutations.	-
Aldoss et al., 2019 [68]	Retrospective study	Median age 68 years, (22–85)	32 adult patients with *TP53*-mutated AML (16 R/R and 15 newly diagnosed)	VEN + HMA	RR = 52% (*n* = 16), with 38% CR/CRi rate in R/R. Lower response = 14% vs. 63%, *p* = 0.025 if prior HMA monotherapy. Higher CR/CRi rate = 78% vs. 41%, *p* = 0.062 if more than 1 *TP53* mutation.	-
Aldoss et al., 2020 [69]	Retrospective study	Median 66 (18–82)	50 adverse-risk AML patients (33 R/R and 17 treatment-naïve) with *FLT3* mutations	VEN-HMA	CR/CRi rate = 60% (*n* = 30) and 42% in R/R AML [37% for patients with prior exposure to *FLT3*-based TKIs and 50% in patients who were naïve for TKIs].	16 (32%) patients developed neutropenic fever, 3 (6%) blood stream bacterial infections, 2 (4%) invasive fungal infection, 2 (4%) grade ≥ 3 bleeding.
Wang et al., 2020 [70]	Retrospective study	Median 63 years (20–88)	40 R/R AML	VEN-based therapy	RR = 50%, with 9 (22.5%) CR/Cri. Higher CR/CRi rate = *NPM1*, *RUNX1*, or *SRSF2* mutations; longer OS = *RUNX1*. Worse outcome = *FLT3-ITD*, *TP53*, or *DNMT3A* mutations.	The most common AE = prolonged cytopenia (67.5% febrile neutropenia).
Piccini et al., 2021 [71,72]	Retrospective analysis	Median 56 (33–74)	47 R/R AML patients	VEN-based regimens	Composite CR rate = 55%, with 16% MRD negative status. Favorable outcome = HSCT-naïve patients and aged >60 years; OS = 10.7 months; longer OS = *NPM1* mutation (median not reached); shorter OS = *NPM1-FLT3/ITD* mutations (2.3 months). 13/24 (54%) patients were successfully bridged to HSCT.	The most common AE = myelosuppression. 100% = grade 4 neutropenia (47/47,) and 95% transfusion-dependent anemia and thrombocytopenia. 21 febrile neutropenia and 17 infectious events were reported (G2, *n* = 10; G3, *n* = 4; G4, *n* = 2). 10 patients experienced oral mucositis, in one case graded >2.
Byrne 2020 [73]	Retrospective study	64.5 years (range 34.5– 73.7 years). 64.5 years (range 34.5– 73.7 years). Median 64.5 (34.5–73.7).	21 post-transplant relapsed AML patients	VEN-based regimens (mainly with HMAs)	RR = 42.1% (*n* = 8, 5 CR and 3 CRi). None of the 4 patients with complex karyotype and *TP53* mutations responded to therapy. Median OS = 7.8 months (range 0.2–12.1 months); significantly longer OS = in patients achieving CR/CRi (*p* = 0.005).	61.9% of patients = infections (7 bacterial pneumonia, 4 sepsis, 4 had fungal pneumonia, 2 oral infections). 9/11 deceased patients had active infections. No patients who achieved a CR/CRi died of infectious complications.
Vigil et al., 2020 [74]	Real-world retrospective cohort	Median age 55.7 years (range 32–73)	9 post allogenic HSCT relapsed AML patients	Low dose VEN (100 mg/day) + decitabine (20 mg/m^2^ day 1–5) (*n* = 8) or azacitidine (75 mg/m^2^ for 7 days)	ORR = 44% (*n* = 4); CRi after the first cycle = 3	-
Zucenka et al., 2021 [75]	Retrospective study	ACTIVE Median 59 (20–71) FLAG-Ida Median 48 (20–75)	49 R/R patients after alloSCT treated	ACTIVE (*n* = 20) or FLAG-Ida (*n* = 29)	ORR 75% ACTIVE vs. 66%FLAG-Ida; *p* = 0.542; CR/CRi rate = 70% ACTIVE vs. 34% FLAG-Ida; *p* = 0.02; OS 13.1 ACTIVE vs. 5.1 months FLAG-Ida; *p* = 0.032. Treatment-related mortality = 0% ACTIVE and 34% FLAG-Ida group (*p* = 0.003).	Febrile neutropenia, catheter-related infections, bacteremia and sepsis = lower in ACTIVE vs. FLAG-Ida. Admission to the ICU during treatment 31% of FLAG-Ida and 5% ACTIVE patients was (*p* = 0.034). No treatment-related deaths in the ACTIVE group compared to 10 in the FLAG-Ida group (*p* = 0.003).
Joshi et al., 2021 [76]	Retrospective analysis	Median 58 (20–72)	29 patients with post allo-HCT AML relapse	VEN regimens as salvage therapy	RR = 38%; including CR/CRi in 8. Median duration of response = 7 months (1–11); median OS = 79 days (2–403), better in responders (403 vs. 55 days, *p* < 0.0001).	Most frequent Grade 3 or 4 toxicities = neutropenia, infections, thrombocytopenia, and anemia. Cycle length adjustments due to hematological toxicity = 19 patients.
Zhao et al., 2021 [77]	Retrospective study	Mean 35.2 ± 11.4	26 patients with AML relapsed after alloHSCT	VEN + azacitidine and donor lymphocyte infusion	ORR = 61.5% (including 26.9% of CRi); median OS = 284.5 days (95% CI: 81–610).	Hematologic AEs occurred in all patients, in particular grade 3/4 agranulocytosis and thrombocytopenia.
Goldberg et al., 2017 [78]	Retrospective single-center analysis	Median age of 66 (29–85)	21 patients with a R/R myeloid malignancy (*n* = 20 AML and *n* = 1 MDS)	VEN + HMA (*n* = 8) or VEN + LDAC (*n* = 16)	ORR = 28.6% (95% CI: 11.3–52.2%) (*n* = 6) [23% for VEN-LDAC, 50% for VEN-azacitidine, and 0% for VEN-decitabine], with 5 CR. No CR in patients who received a prior allogenic HSCT or in those with *FLT3* or *RAS* mutations.	-
Feld et al., 2021 [79]	Retrospective study	Among patients with R/R AML, the median age was 61.5 years	72 patients with AML (including 39 R/R)	VEN + HMA	ORR = 38.5% (*n* = 15/39); CR = 12.8%; CRi = 25.6%. Higher ORR = naïve to HMA (66.7% vs. 14.3% in patients previously exposed). Better response = those harboring *TET2*, *IDH1* or *IDH2* mutations, in contrast to *FLT3* and *RAS* mutations. Median duration of response and median OS = 8.1 months. 8 (20.5%) R/R patients underwent post-treatment allogenic HSCT.	59.1% = grade ≥ 3 infection, 46.5% neutropenic fever, and 71.8% persistent neutropenia
Ganzel et al., 2020 [80]	Retrospective analysis	Median age 67 years, (21–82)	40 adult R/R AML patients	Median daily dose of 400 mg (range 100–800), + HMAs (62.5%) or LDAC (22.5%)	CR/CRi = 37.5% (*n* = 15). Median OS = 5.5 months (6.5 months for patients who survived more than 2 months); shorter survival for patients who underwent prior allogenic HSCT (4.5 months vs. 6 months). 5 patients underwent allogenic HSCT after treatment with VEN.	Gastro-intestinal problems (*n* = 4), infections (*n* = 3), skin complications (*n* = 2), weakness (*n* = 2), and vertigo (*n* = 1).
Gaut et al., 2020 [81]	Retrospective analysis	Median age of 58 years	14 patients with R/R AML (mainly characterized by adverse cytogenetics)	VEN + 8 with azacytidine, 5 with decitabine, and 1 with LDAC	ORR = 35.7% (*n* = 5); CRi = 21.4%; partial remission = 14.3%	100% = grade ≥ 3 neutropenia and thrombocytopenia; 92.9% = grade ≥ 3 anemia. 64.3% febrile neutropenia; 50.0% grade ≥ 3 infection.
Lou et al., 2020 [82]	Retrospective analysis	Median age 61 years (19–73)	48 adult patients with R/R AML	VEN + azacitidine	ORR = 47.9%; CR = 29.2%; CRi = 18.8%. ORR = 75% if *IDH1/2* mutations or *RUNX1* mutation. Median OS = 9.6 months; OS significantly longer = ifCR/CRi (*p* < 0.001) and if *IDH1/2* mutations (not reached vs. 3.2 months in wild-type, *p* < 0.001; HR = 0.069, 95% CI: 0.006–0.726, *p* = 0.026); OS significantly shorter = *TP53* mutation (4.7 months vs. not reached in wild-type, *p* < 0.001; HR = 22.855, 95% CI: 2.549–204.949, *p* = 0.005).	Most common grade 3/4 AEs = neutropenia (91.7%, *n* = 44) and thrombocytopenia (89.6%, *n* = 43). Febrile neutropenia occurred in 40 patients (83.3%).
Morsia et al., 2020 [83]	Retrospective study	Median age of 64.5 years (18–79)	42 R/R AML with the exclusion of post-transplant relapses.	VEN + HMA (mainly decitabine) as salvage therapy	CR/CRi rate = 33.3% (*n* = 14); CR = 19%; CRi = 14.3%. Median OS = 5 months (95% CI: 3–9 months); longer OS if CR/CRi (15 vs. 3 months in non-responders, *p* < 0.001); shorter OS = if *TP53* mutations (*p* = 0.04).	Infections = 85.7%; invasive fungal infections = 9.5% despite azole prophylaxis; heart failure = 19%; renal impairment = 4.8%.
Zappasodi et al. [84]	Retrospective study	From 23 to 67 years	10 heavily pretreated patients with refractory AML	VEN with azacitidine as bridge to allogeneic SCT	ORR = 60%, including 4 CR, 1 CRi, and 1 MLFS. HSCT performed in all 6 responders and in 1 non-responder. Median OS = 8.9 months (range 2–19) and 11.7 months among transplanted patients.	Most frequent AEs = hematological (deep and prolonged grade 3/4 neutropenia, anemia, and thrombocytopenia). 4 infections (3 bacterial infections and 1 invasive aspergillosis), all successfully managed.
Ram et al. 2019 [85]	Retrospective cohort	Median 76 (41–92)	23 patients AML refractory to HMAs–including also patients relapsed after allogenic HSCT	VEN + LDAC (*n* = 4), azacitidine (*n* = 13), azacitidine and donor lymphocyte infusions (*n* = 5), and donor lymphocyte infusions alone (*n* = 1)	CR/CRi = 43%. 6- and 12-month OS rate = 74% and 25%, respectively. Median OS = 5.6 months (95% CI: 4.9–6.2), Median OS in responders = 10.8 months, 95% CI: 6.2–15.4 Median OS in non-responders = 2.8 months, 95% CI: 0.9–4.8, (*p* < 0.001). CR/CRi = 67% in those relapsed after allogeneic HSCT (*n* = 6) with a median OS of 12.4 months.	Febrile neutropenia = 78% of patients.
Tiong et al., 2021 [86]	Retrospective study	5 patients age 59–79; 7 patients age 25–81.	12 patients with R/R AML carrying the *NPM1* mutation (5 with molecular persistence and 7 with progression or relapsed)	VEN + LDAC or azacitidine	CR with MRD negativity = 92% after 1–2 cycles. Median relapse-free survival = not reached in the 5 responders with previous molecular persistence (median follow-up = 20 months).	Most common AEs = grade 4 neutropenia (*n* = 8, 67%), grade 4 thrombocytopenia (*n* = 5, 42%), and febrile neutropenia (*n* = 2, 17%) associated with 1 invasive fungal infection and 1 grade 4 lung infection.
Stahl et al., 2021 [87]	Retrospective study	Median 67 (29–86)	Large cohort of real-world patients (*n* = 86) with R/R AML	VEN + HMA or LDAC	ORR = 31%; CR/CRi rate = 24%. RR = 49% in patients treated with VEN + azacitidine or decitabine vs. 15% in those treated with VEN + LDAC; OR = 5.43, 95% CI: 1.55–19, *p* = 0.008; 25% vs. 15%; OR = 1.92, 95% CI: 0.44–8.31, *p* = 0.38. Median duration of response = 7.8 months; median OS = 6.1 months (95% CI: 4.9–10 months); median OS = 25 months in those treated with VEN + azacitidine (), compared to VEN + decitabine (5.4 months) (*p* = 0.13) and VEN + LDAC (3.9 months) groups (*p* = 0.003). Higher response = if *NPM1* mutations; lower response = if adverse cytogenetics and mutations in *TP53*, *KRAS/NRAS* and *SF3B1*.	-
Tenold et al., 2021 [13]	Retrospective study	Median 57 (range 25–86)	A small cohort (*n* = 25) of real-world patients with R/R AML	VEN + decitabine or azacitidine	ORR = 52%, with 4 achieving CR (16%), 4 CRi (16%), and 5 MLFS (20%). Median OS = 5.5 months (95% CI: 2.9–21.6), significantly longer for patients achieving CR/CRi (21.6 months, 95% CI: 15.2-not reached; *p* < 0.0026). 1-year OS rate = 100% for patients reaching CR/CRi, higher than for patients with MLFS (0%) or no responders (14%).	Most common AEs = febrile neutropenia (*n* = 10, 40%) and prolonged pancytopenia (*n* = 9, 36%).
Tong et al., 2021 [88]	Retrospective study	Median 47.5 (12–84)	22 heavily pre-treated R/R-AML (8 relapsed AML including 2 after HSCT and 14 primary refractory AML including)	VEN + decitabine	ORR = 45.5%; 4 patients relapsed with a median time of 5 months. 1-year OS rate = 31.8%, including 59.1% in those achieving CR/CRi and 10.4% in non-responders (*p* = 0.001).	All patients had grade 4 neutropenia and thrombocytopenia.
Maiti et al., 2021 [89]	Retrospective study	DEC10-VEN median 64 (18–85); IC cohort 58 (19–80).	65 R/R patients compared to 130 patients receiving IC	10-day decitabine + VEN	ORR = 60% DEC10-VEN vs. 36% IC cohort; OR 3.28; *p* < 0.001; CRi = 19% DEC10-VEN vs. 6% IC cohort; OR, 3.56; *p* = 0.012; MRD negativity = 28% DEC10-VEN vs. 13% IC cohort; OR, 2.48; *p* = 0.017; median event-free survival = 5.7 months DEC10-VEN vs. 1.5 IC cohort; hazard ratio [HR], 0.46; 95% CI, 0.30–0.70; *p* < 0.001; median OS = 6.8 months DEC10-VEN vs. 4.7IC cohort; HR, 0.56; 95% CI, 0.37–0.86; *p* = 0.008.	Nonhematologic grade 3/4 TEAEs = 21 patients with IC and 45 receiving DEC10-VEN (*p* = 0.628). Grade 3/4 infections = 25 patients in the DEC10-VEN group and 5 patients in the IC group (*p* = 0.057). Febrile neutropenia = 30% in the DEC10-VEN cohort and in the IC cohort (*p* = 0.101).
Masarova et al., 2021 [90]	Retrospective study	Median 69 (46–80)	14 naïve and 17 R/R post–MPN-AML patients AML post myeloproliferative neoplasms	VEN regimens	No responses. Median OS = 3 months.	Most frequent AEs = prolonged cytopenias and infections.
Amit et al., 2021 [91]	Retrospective study	Median 65 (41–75)	22	VEN + donor lymphocyte infusion	RR = 50% (9 CR/CRi, 2 MLFS). Median time to response = 28 days (18–67); duration of response = 135 days (31–564). Median OS = 6.1 months (95% CI 0.73–11.4).	50% = gastrointestinal toxicity (grade 1–2 diarrhea); 73% = hematological toxicity that resulted in complete discontinuation of azacitidine or dose reduction of VEN.
Moukalled et al., 2019 [92]	Case report/case series	48-years 27-year 34-year 45-year	4 cases of heavily pretreated R/R AML patients	VEN-based regimens	Stable disease for around 4 months in patient 1 (a female with *NPM1* mutation and de novo *FLT3 ITD* relapsed after haploidentical-SCT and treatment with azacitidine + sorafenib and 2 doses of donor lymphocyte infusion); complete molecular remission in patient 2 (a male with *inv (16)* relapsed after matched-related-allogenic SCT and azacitidine treatment and refractory to donor lymphocyte infusion); No response in the other 2 patients—a male with *FLT3-TKD* mutation and a male with AML-myelomonocytic features, both relapsed after SCT.	1 patient developed a suspected skin GVHD; 1 a grade 3 cytopenia (mainly neutropenia) requiring intermittent interruptions of VEN; 2 developed fatal infections with multiorgan failure.
Andreani et al., 2019 [93]	Case report/case series	60-years	Patient with *NPM1*-, *IDH1*-, and *FLT3cod835*-mutation, relapsed after alloSCT and refractory to a new re-induction chemotherapy	VEN plus decitabine	Responded to combined treatment of with persistent molecular remission.	Cytopenia, infection of central venous catheter, sepsis.

ACTIVE = VEN + LDAC + actinomycin D; AEs = adverse events; AlloSCT = allogeneic stem cell transplantation; CR = complete response; CRi = CR with incomplete blood count recovery; FLAG-Ida = fludarabine + cytarabine + idarubicin; GVHD = graft-vs.-host-disease; HMA = hypomethylating agent; HSCT = hematopoietic stem cell transplantation; IC = intensive chemotherapy; LDAC = low-dose cytarabine; MDS = myelodysplastic syndrome; MLFS = morphological leukemia free state; MRD = minimal residual disease; OR = odds ratio; ORR = objective response rate; OS = overall survival; R/R AML = relapsed/refractory acute myeloid leukemia; RR = response rate; TEAEs = treatment-emergent adverse events; TKIs = tyrosine kinase inhibitors; VEN = venetoclax.

## Supplementary Materials

The following are available online at https://www.mdpi.com/article/10.3390/cancers14010022/s1, Table S1: Ongoing clinical studies.
