# Supplementary material for: Venetoclax in Relapsed/Refractory Acute Myeloid Leukemia: Are Supporting Evidences Enough?
Source: Cancers (Basel). 2021 Dec 21;14(1):22. doi: 10.3390/cancers14010022 (PMC8750253; doi:10.3390/cancers14010022)
Supplement: Supplementary file 1 [file cancers-14-00022-s001.zip › cancers-1448391-supplementary.pdf]

cancers MDPI

Supplementary Files

Venetoclax in Relapsed/Refractory Acute Myeloid Leukemia: Are Supporting Evidences Enough?

Serena Brancati, Lucia Gozzo, Giovanni Luca Romano, Calogero Vetro, Ilaria Dulcamare,Cinzia Maugeri, Marina Parisi, Laura Longo, Daniela Cristina Vitale, Francesco Di Raimondo and Filippo Drago

Table S1. Ongoing clinical studies (www.clinicaltrial.gov; update on 31 October 2021).

ID	Title	Trial design	Age (years)	Number of Estimated patients (disease)	Arms and Interventions	Primary Outcome Measures	Follow-Up Duration	Start Date/ Estimated Study Completion Date
NCT04330820	Trial for Relapsed or Refractory AML Patients Combining Cytarabine and Mitoxantrone With Venetoclax (RELAX)	Phase I/II, open-label Phase I dose-escalation study, Sequential Assignment	18-75	60	Venetoclax+Cytarabin+ Mitoxantron<br>The treatment plan combines a fixed dose of venetoclax and mitoxantrone with increasing doses of cytarabine (V-MAC).	Maximum tolerated dose (=recommended phase II dose) of cytarabine in combination with venetoclax plus mitoxantrone CR/CRi rate	48 months	April 2020/April 2024
NCT03867682	Venetoclax and Lintuzumab-Ac225 in AML Patients	Phase I/II, Open Label, Single Group	18 and older	38	Lintuzumab-Ac225 administered on Day 1 of each cycle for four cycles (unless in the 0.5 µCi/kg or 0.25 µCi/kg cohorts, where there is a potential for an additional four cycles, pending PI and Medical Monitor review).<br>Venetoclax taken on Days 1-21 of each cycle for up to 12 cycles.<br>Each cycle is 28 days, with a potential to expand to 42 days to allow for full hematologic recovery.	Phase I: Maximum Tolerated Dose (MTD) of Lintuzumab-Ac225<br><br>Phase II: Overall Response (CR + CRh)	2 years	January, 2020/January 2023
NCT03932318	Venetoclax, Azacitidine, and Lintuzumab-Ac225 in AML Patients	Phase I/II trial, Open Label, Single Group	≥18	38	Lintuzumab-Ac225 will be administered on Day 8 of each cycle for four cycles (unless in the 0.5 µCi/kg or 0.25 µCi/kg cohorts, where there is a potential for an additional four cycles, pending PI and Medical Monitor review).<br>Venetoclax will be taken on Days 1-21 of each cycle for up to 12 cycles.<br>Azacitidine will be administered on Days 1-7 of each cycle for up to 12 cycles.<br>Each cycle is 28 days, with a potential to expand to 42 days to allow for full	Phase I: Maximum Tolerated Dose (MTD) of Lintuzumab-<br><br>Phase II: Overall Response (CR + CRh + CRi + MLFS)	2 years	September 2021/ September 2024

					hematologic recovery.			
NCT04755244	A Study of ALX148 With Venetoclax and Azacitidine for Acute Myeloid Leukemia (ASPEN-05)	Phase I/II trial, Open Label, Single Group	18 and older	97	ALX148 + venetoclax + azacitidine<br>Phase 1a: Participants will receive escalating doses of ALX148 in combination with venetoclax and azacitidine<br><br>Phase 1b/2: Participants will receive ALX148 at the recommended Phase 2 dose in combination with venetoclax and azacitidine	Phase 1: Dose Limiting Toxicities (DLT)<br><br>Phase 2: Composite complete remission rate (CRc)	6 months	April 2021/ December 2023
NCT04267081	Study of Venetoclax in Combination With Azacytidine in AML Patients Selected Using Ex Vivo Drug Sensitivity Screening (VenEx)	Phase II, multicenter two-stage, two-arm, open label, non-randomized, parallel assignment	18-100	100	All patients in validation and study cohorts (ARM1 and ARM2) will receive azacytidine and venetoclax. The purpose for the validation cohort is to validate the specificity and sensitivity of the ex vivo drug testing. Patients exhibiting ex vivo sensitivity and receiving azacytidine-venetoclax in validation cohort are analyzed also for study cohort.	Complete remission (CR)/complete remission rate with incomplete hematologic recovery (CRi) rate in study cohort after three cycles.	3 years	February 2020/ February 2024
NCT04070768	Study of the Safety and Efficacy of Gemtuzumab Ozogamicin (GO) and Venetoclax in Patients With Relapsed or Refractory CD33+ Acute Myeloid Leukemia:Big Ten Cancer Research Consortium BTCRC-AML17-113	Phase Ib, Single Group Assignment, Open Label	18 and older	24	Gemtuzumab Ozogamicin 3mg/m^2, Days 1,4,7 Venetoclax, 100,200,400, or 600mg Daily Dose	Maximum Tolerated Dose (MTD) of Venetoclax when administered with GO	7 months	September 2019/ October 2022
NCT04017546	CYC065 CDK Inhibitor and Venetoclax Study in Relapsed/Refractory AML or MDS	Phase I, open-label, single arm, dose escalation study	12 and older	25 (AML or MDS)	CYC065 will be administered intravenously via 4-hour infusion on Day 1 and Day 15. Venetoclax will be taken daily on Day 1 through Day 15. One cycle will be 28 days or 4 weeks.	Maximum tolerated dose (MTD)	28 days	July, 2019/ December 2020
NCT03625505	A Study to Assess Safety and Efficacy of Venetoclax in Combination With Gilteritinib in Participants With Relapsed/Refractory Acute Myeloid Leukemia	Phase I, Non-Randomized, open-label, sequential assignment	18 and older	61	Different combinations of dose levels for venetoclax in combination with gilteritinib will be administered to determine the recommended phase 2 dose (RPTD).	Recommended Phase 2 Dose (RPTD) of Co-administered Study Drugs	6 months after the last participant is enrolled	October 2018/ November 2021
NCT02781883	Clinical Trial of BP1001 in Combination With Venetoclax Plus Decitabine in AML	Phase IIa, Open-label, Non-Randomized, Parallel Assignment	18 and older	108 (including both untreated and R/R)	BP1001 in combination with Ventoclax plus decitabine	Assessment of efficacy in untreated AML subjects by bone marrow aspirate or biopsy	180 days	May 2016/ December 2021

						Assessment of efficacy in refractory/relapsed AML subjects by bone marrow aspirate or biopsy		
NCT03484520	A Study of Venetoclax and Dinaciclib (MK7965) in Patients With Relapsed/Refractory Acute Myeloid Leukemia	Phase 1b, non-randomized, single group assignment	18 and older	48	Venetoclax and dinaciclib will be administered in combination. Different combinations of dose levels for venetoclax and dinaciclib will be explored.	Pharmacokinetic parameters Recommended Phase 2 Dose (RPTD) of co-administered Dinaciclib and Venetoclax	29 days	July 2018/ December 2021
NCT03940352	HDM201 in Combination With MBG453 or Venetoclax in Patients With Acute Myeloid Leukemia (AML) or High-risk Myelodysplastic Syndrome (MDS)	Phase 1b, non-randomized, open label, parallel assignment	18 and older	80 (including R/R AML, untreated AML and high-risk MDS)	Treatment arm1: HDM201+MBG453 Treatment arm2: HDM201+venetoclax	Incidence and severity of Adverse Events (AEs) and Serious Adverse Events (SAEs) as a measure of safety Incidence of dose limiting toxicities (DLTs) of treatment Frequency of dose interuptions, dose reductions, dose intensities	24 months	June 2019/ December 2021
NCT03113643	SL-401 in Combination With Azacitidine or Azacitidine/Venetoclax in Acute Myeloid Leukemia (AML), High-Risk Myelodysplastic Syndrome (MDS) or Blastic Plasmacytoid Dendritic Cell Neoplasm (BPDCN)	Phase 1b, non-randomized, open label, single group assignment	18 and older	72 (including R/R AML, untreated AML and high-risk MDS)	SL-401 will be administered every 4 weeks, on a 28 day cycle; SL-401 will be given intravenously; Azacitidine will be administered every 4 weeks, on a 28 day cycle; Azacitidine will be given intravenously or subcutaneously; Venetoclax will be administered for 21 days on a 28 day cycle; Venetoclax will be taken orally	Maximum Tolerated Dose	2 years	June 2017/ May 2024
NCT04086264	IMGN632 as Monotherapy or With Venetoclax and/or Azacitidine for Patients With CD123-Positive Acute Myeloid Leukemia	Phase 1b/2, non-randomized, open label, parallel assignment	18-120	274 (including R/R AML, untreated AML)	Experimental: Regimen A IMGN632, administered intravenously on Day 7 of a 28 day cycle at 0.015 mg/kg, 0.045 mg/kg, or 0.09 mg/kg, in combination with azacitidine, administered subcutaneously or intravenously daily at 75 mg/m2 on Days 1 to 7 of a 28 day cycle. Cycle 1 azaciditine dose in subsequent cohorts may be reduced. Experimental: Regimen B IMGN632, administered intravenously on Day 7 of a 21 day cycle at 0.015 mg/kg, 0.045 mg/kg, or 0.09 mg/kg, in combination with venetoclax, administered orally daily at 100 mg on Day 1, 200mg on Day 2, and 400 mg on the day 3 up to Day 21	Safety and Tolerability Preliminary antileukeumia activity Minimal Residual Disease Levels	20 months	November 2019/ June 2022

					of a 21 day cycle. Alternate schedules with reduced venetoclax administration may be explored. Experimental: Regimen C IMGN632, administered intravenously on Day 7 of a 28 day cycle at 0.015 mg/kg, 0.045 mg/kg, or 0.09 mg/kg, in combination with azacitidine, administered subcutaneously or intravenously daily at 35-75 mg/m2 given for Days 1 to 7 of a 28 day cycle and **venetoclax**, administered orally daily at 100 mg on Day 1, 200mg on Day 2, and 400 mg on Day 3 up to Day 28 of a 28 day cycle. Alternate schedules with reduced **venetoclax** administ ration or reduced azacitidine dose or administration may be explored.			
NCT04666649	Pegcrisantaspase in Combination With Venetoclax for Treatment of Relapsed or Refractory Acute Myeloid Leukemia (R/R AML)	Phase 1, non-randomized, open label, sequential assignment	18 and older	30	Cohort1: The subject will take 400mg of Venetoclax every day as a pill by mouth and a dose of 500 IU/m² of Pegcrisantaspase in an IV every 14 days (per cycle) Cohort 2: The subject will take 400mg of Venetoclax every day as a pill by mouth and a dose of 750 IU/m² of Pegcrisantaspase in an IV every 14 days (per cycle) Cohort 3: The subject will take 400mg of Venetoclax every day as a pill by mouth and a dose of 1000 IU/m ² of Pegcrisantaspase in an IV every 14 days (per cycle) Cohort 4: The subject will take 600mg of Venetoclax every day as a pill by mouth and a dose of 1000 IU/m ² of Pegcrisantaspase in an IV every 14 days (per cycle)	Incidence of regimen limiting toxicities (RLTs) and treatment-emergent adverse events (TEAE)	1 year	January 2021/ July 2022
NCT03672695	Phase I Dose Escalation Study of Intravenously Administered S64315 in Combination With Orally Administered Venetoclax in Patients With Acute Myeloid Leukaemia.	Phase 1, open label, sequential assignment	18 and older	40	The treatment combination period can only begin after the planned dose of venetoclax is reached. Depending on the administration dosing schedule, the combination treatment at the planned doses may be preceded by a 2-week Lead-In Dose period of S64315 (fixed dose) during which the patient continues to receive venetoclax daily.	Incidence of Dose Limiting Toxicity (DLTs) Incidence and severity of AEs and SAEs Number of participants with dose interruptions, dose reductions	6 months	November 2018/ September 2022

					Once the planned dose of both drugs is reached the schedule will be a 21-day cycle with a weekly regimen for S64315 and a daily regimen for venetoclax. S64315 should be administered 2 to 4 hours after venetoclax intake, via IV infusion. The dose escalation will start at 50 mg once a week and doses up to 250 mg once a week might be explored. Venetoclax will be administered orally once a day. The dose escalation will start at 100 mg daily and doses up to 600 mg daily might be explored. Venetoclax must be taken with a meal (ideally during breakfast) in order to avoid reduced efficacy.			
NCT04172844	Pevonedistat, Azacitidine, and Venetoclax for the Treatment of Patients With Acute Myelogenous Leukemia (PAVE)	Phase 1b, non-randomized, open label, sequential assignment	18 and older	24 (only R/R in the escalation; both R/R and untreated in the dose expansion)	Dose Escalation<br>This study uses a varied 3 + 3 design. Three patients will be started at a dose of 10 mg/m^2 days 1, 3 and 5. If no DLTs are observed in the first 3 participants, then a new cohort will be enrolled at the next planned dose level of 15 mg/m^2 days 1, 3 and 5. If two out of three subjects experience a DLT, then they will de-escalate one dose level. If one subject in three experiences a DLT, then expand up to three subjects at 20 mg/m^2 day 1, 3 and 5. If two out of six subjects experience a DLT, de-escalate one level. All subjects will receive Azacitidine and Venetoclax at the following dosages and timing:<br>• 75mg/m^2 Days 1-7 given IV<br>• 100 mg on cycle 1 day 1, 200 mg daily on cycle 1 day 2, 400 mg on cycle 1 day 3 and thereafter from cycle 1. Venetoclax is given for a minimum of 21 days and a maximum of 28 days.)<br>Dose Expansion Phase<br>Patients will receive the recommended phase 2 dose (RP2D) identified from dose-escalation phase.	Recommended phase 2 dose of pevonedistat when co-administered with azacitidine and venetoclax in patients with AML<br>The toxicity profile of pevonedistat, azacitidine, and venetoclax combination therapy	5 years	January 2020/ December 2025
NCT0373587	Venetoclax and	Phase Ib/II,	18 and	32	Patients receive quizartinib	Maximum tolerated	5 years	January

5	Quizartinib in Treating Patients With FLT3-mutated Recurrent or Refractory Acute Myeloid Leukemia	open label, single group assignment	older		PO QD on days 1-28 and venetoclax PO QD beginning on day 8 of cycle 1. Treatment repeats every 28 days for up to 24 cycles in the absence of disease progression or unacceptable toxicity. Patients may continue treatment beyond 24 cycles at the discretion of the treating physician.	dose (MTD) as determined by dose limiting toxicity (Phase Ib) Recommended phase II dose as determined by MTD (Phase Ib) Composite complete remission rate (CRc) rate		2019/ December 2021
NCT0366272 4	Venetoclax Registry (VENreg)	Observational (Cohort study)	16 and older	100	Registry study observing clinical and biological characteristics of patients with acute myeloid leukemia who are treated with Venetoclax.	Overall response rate of Venetoclax treatment	5 years	November 2017/ November 2025
NCT0443569 1	Magrolimab, Azacitidine, and Venetoclax for the Treatment of Acute Myeloid Leukemia	Phase Ib/II, open label, single group assignment	18 and older	38	Patients receive azacitidine SC or IV over 30-60 minutes on days 1-7, venetoclax PO QD on days 1-28 of cycle 1 (may be reduced to days 1-21 for subsequent cycles after principal investigator approval), and magrolimab IV over 2-3 hours on days 1, 4, 8, 11, 15, and 22 of cycle 1, days 1, 8, 15, and 22 of cycle 2, and days 1 and 15 of cycle 3 and subsequent cycles. Treatment repeats every 28 days for up to 12 cycles in the absence of disease progression or unacceptable toxicity.	Maximum tolerated dose of the combination drugs (phase Ib) Response rate (phase II) Incidence of adverse events (phase II) Event-free survival (phase II) Duration of response (phase II) Overall survival (phase II)	100 days	July 2020/ December 2021
NCT0362917 1	Liposome-encapsulated Daunorubicin-Cytarabine and Venetoclax in Treating Participants With Relapsed, Refractory or Untreated Acute Myeloid Leukemia	Phase II, open label, single group assignment	18 and older	52 (including R/R and untreated)	INDUCTION: Participants receive liposome-encapsulated daunorubicin-cytarabine IV over 90 minutes on days 1, 3, and 5 of course 1 and on days 1 and 3 of course 2. Participants also receive venetoclax PO QD on days 2-21. Treatment repeats every 28 days for up to 2 courses in the absence of disease progression or unacceptable toxicity. CONSOLIDATION: Participants receive liposome-encapsulated daunorubicin-cytarabine IV over 90 minutes on days 1 and 3 and venetoclax PO QD on days 2-21. Treatment repeats every 28 days for up to 4 courses in the absence of disease progression or unacceptable toxicity.	Achievement of composite complete remission Incidence of adverse events	1 year	October 2018/ June 2022
NCT0474623 5	Venetoclax and ASTX727 for the	Phase 2, open label, single	18 and older	40 (including	Patients receive decitabine and cedazuridine PO daily on	Overall response rate (ORR)	5 years	February 2021/

	Treatment of Relapsed, Refractory, or Newly Diagnosed Acute Myeloid Leukemia	group assignment		R/R and untreated)	days 1-5 and venetoclax PO daily on days 1-28 of the first cycle and on days 1-21 of subsequent cycles. Treatment repeats every 28 days for up to 24 cycles in the absence of disease progression or unacceptable toxicity.			October 2022
NCT03063944	STAT Inhibitor OPB-111077, Decitabine and Venetoclax in Treating Patients With Acute Myeloid Leukemia That Is Refractory or Newly Diagnosed and Ineligible for Intensive Chemotherapy	Phase I, open label, single group assignment	18 and older	59 (both R/R and untreated)	Patients receive STAT inhibitor OPB-111077 PO QD on days 1-28. Courses repeat every 28 days in the absence of disease progression or unacceptable toxicity. Patients also receive decitabine IV on days 8-12. Treatment repeats every 28 days for up to 2 courses in the absence of disease progression or unacceptable toxicity. Venetoclax given PO	Incidence of grade 4, non-hematologic dose limiting toxicities	2 years	March 2017/ November 2023
NCT03874052	Ruxolitinib and Venetoclax in Treating Patients With Relapsed or Refractory Acute Myeloid Leukemia	Phase I, open label, single group assignment	18 and older	30	Patients receive ruxolitinib PO BID and venetoclax PO QD on days 1-28. Treatment repeats every 28 days for up to 2 cycles in the absence of disease progression or unacceptable toxicity. Patients may receive additional cycles of ruxolitinib and venetoclax at the discretion of the sponsor-investigator.	Dose-limiting toxicities (DLT) of ruxolitinib and venetoclax in combination	24 months	August 2019/ December 2022
NCT03194932	Study of Venetoclax in Combination With Chemotherapy in Pediatric Patients With Refractory or Relapsed Acute Myeloid Leukemia or Acute Leukemia of Ambiguous Lineage	Phase I, open label, single group assignment	2-20	72	In Part 1, venetoclax with cytarabine will initially be given at dose level 1 and escalated based on tolerability. Idarubicin will be given only at dose level 4. Two expansion cohorts will be enrolled: Cohort A will be a group of 12 participants receiving the recommended phase 2 doses (RP2D) of venetoclax plus cytarabine. Cohort B will be a group of 12 participants receiving the RP2D of venetoclax plus cytarabine and idarubicin. Intrathecal Triple Therapy (ITMHA) will be given prior to cycle 1. Patients without evidence of central nervous system (CNS) leukemia will receive no further IT therapy during cycle 1. Patients with CNS disease will receive weekly ITMHA beginning on day 8 until the cerebrospinal fluid becomes free of leukemia.	Maximum tolerated combination (MTC)	6 months	July 2017/ October 2021

					Cohort C: Participants will receive venetoclax PO on days 1-21, azacitidine IV on days 1-7, and cytarabine Q12H on days 8-11.			
NCT03214562	Venetoclax With Combination Chemotherapy in Treating Patients With Newly Diagnosed or Relapsed or Refractory Acute Myeloid Leukemia	Phase 1b/2 open label, single group assignment	18 and older	116 (both R/R and untreated)	venetoclax, FLAG-IDA	Overall response rate (ORR)<br>CR/CRi rate<br>Hematologic response<br>Duration of response<br>Event-free survival<br>Overall survival	6 years	September 2017/ September 2023
NCT03661307	Quizartinib, Decitabine, and Venetoclax in Treating Participants With Untreated or Relapsed Acute Myeloid Leukemia or High Risk Myelodysplastic Syndrome	Phase 1/2 open label, single group assignment	18 and older	52 (including R/R, untreated AML, MDS)	Patients receive decitabine IV over 1 hour on days 1-10, quizartinib PO every day beginning on day 1 of cycle 1, and venetoclax PO on days 1-14 (days 1-21 if persistent leukemia). Cycles repeat every 28 days in the absence of disease progression or unacceptable toxicity.	Maximum tolerated dose of the combination drugs (Phase I)<br>Incidence of adverse events and Overall response rate (ORR) (Phase II)	3 months	October 2018/ January 2022
NCT03390296	OX40, Venetoclax, Avelumab, Glasdegib, Gemtuzumab Ozogamicin, and Azacitidine in Treating Patients With Relapsed or Refractory Acute Myeloid Leukemia	Phase Ib/II, non-randomized, open label, parallel assignment	18 and older	138	Arm A (anti-OX40 antibody PF-04518600)<br>Patients receive anti-OX40 antibody PF-04518600 IV over 60 minutes on days 1 and 14. Cycles repeat every 28 days in the absence of disease progression or unacceptable toxicity.<br>Arm B (azacitidine, venetoclax, GO)<br>Patients receive azacitidine IV over 10-40 minutes or via injection SC on days 1-7 or 1-5 and 8-9. Patients also receive venetoclax PO on days 1-28 and GO IV over 2 hours on day 8. Cycles repeat every 28 days in the absence of disease progression or unacceptable toxicity.<br>Arm C (azacitidine, GO, avelumab)<br>Patients receive azacitidine and GO as in Arm B. Patients also receive avelumab IV over 60 minutes on days 1 and 14. Cycles repeat every 28 days in the absence of disease progression or unacceptable toxicity.<br>Arm D (azacitidine, venetoclax, avelumab)<br>Patients receive azacitidine and venetoclax as in Arm A and avelumab as in Arm C. Cycles repeat every 28 days	Incidence of adverse events (Arms A-F)<br>Composite complete response (CRc) (Arms B-F)	5 years	December 2017/ December 2024

					in the absence of disease progression or unacceptable toxicity.<br>Arm E (azacitidine, avelumab, anti-OX40 antibody PF-04518600)<br>Patients receive azacitidine and avelumab as in Arm C and anti-OX40 antibody PF-04518600 as in Arm A. Cycles repeat every 28 days in the absence of disease progression or unacceptable toxicity.<br>Arm F (GO, glasdegib)<br>Patients receive GO IV over 2 hours on days 1, 4, and 7, and glasdegib PO on days 1-28. Cycles repeat every 28 days in the absence of disease progression or unacceptable toxicity.			
NCT03218683	Study of AZD5991 Alone or in Combination With Venetoclax in Relapsed or Refractory Haematologic Malignancies.	Phase 1/1b/2a, 3-Part, Open-Label, Multicentre Stud, single group assignment	18-85	121	Monotherapy AZD5991<br>Dose escalation - multiple dose levels<br>Monotherapy AZD5991 expansion<br>Dose expansion AZD5991 + venetoclax<br>Dose escalation - multiple dose levels	Incidence of Adverse Events<br>Dose limiting toxicities<br>maximum tolerated dose	12 months	August 2017/ November 2022
NCT03634228	Milademetan Tosylate and Low-Dose Cytarabine With or Without Venetoclax in Treating Participants With Recurrent or Refractory Acute Myeloid Leukemia	Phase I/II, Open-Label, parallel assignment	18 and older	58 (including R/R and untreated)	Arm A (low dose cytarabine, MDM2 inhibitor DS-3032b)<br>Patients receive low dose cytarabine SC BID on days 1-10 and milademetan tosylate PO QD on days 8-14, 8-21, or 5-7 and 15-17. Cycles repeat every 28 days in the absence of disease progression or unacceptable toxicity.<br>Arm B (low dose cytarabine, MDM2 inhibitor DS-3032b)<br>Patients receive low dose cytarabine SC BID on days 1-10, milademetan tosylate PO QD on days 8-14, 8-21, or 5-7 and 15-17, and venetoclax PO QD on days 1-14. Cycles repeat every 28 days in the absence of disease progression or unacceptable toxicity.	Incidence of adverse events (Phase I)<br>Maximum tolerated dose (MTD) (Phase I)<br>Overall response rate (Phase II)	1 year	December 2018/ May 2022
NCT04797767	Venetoclax and CLAG-M for the Treatment of Acute Myeloid Leukemia and High-Grade Myeloid Neoplasms	Phase I, Open-Label, single group assignment	18 and older	20 (both R/R and untreated AML)	Treatment (CLAG-M, venetoclax)<br>Patients will receive induction with granulocyte colony-stimulating factor on days 0-5 (if peripheral white blood cell count is less than 20,000/uL), cladribine on	Incidence of adverse events<br>Maximum tolerated dose of venetoclax in combination with CLAG-M	12 months	May 2021/ December 2024

					days 1-5, cytarabine on 1-5, and mitoxantrone on days 1-3. Patients also receive venetoclax orally (PO) on days 1-14. Treatment repeats every 28-35 days for up to 2 induction cycles including mitoxantrone, and up to 4 consolidation cycles without mitoxantrone in the absence of disease progression or unacceptable toxicity.			
NCT04752163	DS-1594b With or Without Azacitidine, Venetoclax, or Mini-HCVD for the Treatment of Relapsed or Refractory Acute Myeloid Leukemia or Acute Lymphoblastic Leukemia	Open-Label Phase 1/2, Non-Randomized, parallel assignment	18 and older	122	Cohort A and B (DS-1594b) Patients with MLLr or NPM1m receive DS-1594b PO BID on days 1-28. Cycles repeat every 28 days for up to 2 years in the absence of disease progression or unacceptable toxicity. Cohort C (DS-1594b, venetoclax, azacitidine) Patients receive DS-1594b PO BID on days 1-28, venetoclax PO QD on days 1-28, and azacitidine IV or SC on days 1-7. Cycles repeat every 28 days for up to 2 years in the absence of disease progression or unacceptable toxicity. Cohort D (DS-1594b, mini-HCVD) Patients receive DS-1594b PO BID on days 1-28. Food-Effect (DS-1594b) Patients receive DS-1594b PO BID on days 1-8 within 30 minutes after eating a standard meal and PO BID on days 9-15 under fasting conditions in the absence of disease progression or unacceptable toxicity. Phase I (DS-1594b) Patients receive DS-1594b PO BID on days 1-28 in the absence of disease progression or unacceptable toxicity.	Maximum tolerated dose (MTD) (Phase I) Recommended phase 2 dose (RP2D) (Phase I) Incidence of adverse events (Phase I) Complete remission (CR) and complete remission with partial hematologic recover (CRh) rate (Phase II)	2 years	July 2021/ November 2022
NCT03404193	Venetoclax and Decitabine in Treating Participants With Relapsed/Refractory Acute Myeloid Leukemia or Relapsed High-Risk Myelodysplastic Syndrome	Phase II Study, open label, single assignment	18 and older	400	Participants receive decitabine IV over 1 hour on days 1-10 and may also receive decitabine on days 1-5 after achieving complete remission/complete remission with incomplete count recovery during consolidation/maintenance. Participants also receive venetoclax PO daily on days 1-28 of cycle 1 and on days	Overall response rate (ORR)	5 years	January 2018/ December 2024

					1-21 of subsequent cycles. Treatment repeat every 28 days for up to 24 cycles in the absence of disease progression or unacceptable toxicity.			
NCT04140487	Azacitidine, Venetoclax, and Gilteritinib in Treating Patients With Recurrent/Refractory FLT3-Mutated Acute Myeloid Leukemia, Chronic Myelomonocytic Leukemia, or High-Risk Myelodysplastic Syndrome/Myeloproliferative Neoplasm	Phase I/II, open label, single group assignment	18 and older	42	Patients receive azacitidine SC or IV over 30-60 minutes on days 1-7, venetoclax PO QD on days 1-28 of cycle 1 and on days 1-21 of subsequent cycles, and gilteritinib PO QD on days 1-28. Treatment of azacytidine and venetoclax repeats every 28 days for up to 24 cycles in the absence of disease progression or unacceptable toxicity. Cycles of gilteritinib repeat every 28 days in the absence of disease progression or unacceptable toxicity.	Maximum-tolerated dose (MTD) of gilteritinib (Phase I) Overall response rate (OR) (Phase II)	3 years	December 2019/ September 2024
NCT04487106	Azacitidine, Venetoclax, and Trametinib for the Treatment of Relapsed or Refractory Acute Myeloid Leukemia or Higher-Risk Myelodysplastic Syndrome	Phase II, open label, single group		40	INDUCTION (CYCLE 1): Patients receive azacitidine IV over 30-60 minutes or SC on days 1-7, venetoclax PO QD on days 1-28, and trametinib PO QD on days 1-28 in the absence of disease progression or unacceptable toxicity. CONSOLIDATION (CYCLES 2-24): Patients receive azacitidine IV over 30-60 minutes or SC on days 1-7, venetoclax PO QD on days 1-21, and trametinib PO QD on days 1-28. Treatment repeats every 28 days for up to 23 cycles in the absence of disease progression or unacceptable toxicity.	Overall survival (Cohort A) Complete remission(CR)/complete remission without recovery of counts (CRi) (Cohort B)	3 years	July 2020/ June 2024
NCT04146038	Salsalate, Venetoclax, and Decitabine or Azacitidine for the Treatment of Acute Myeloid Leukemia or Advanced Myelodysplasia/Myeloproliferative Disease	Phase 2, open label, single group assignment	18 and older	20 (including R/R, untreated AML, MDS)	CYCLE 1: Patients receive salsalate PO BID until completion of cycle 1. 24-48 hours later or concurrent with salsalate, patients begin to receive decitabine IV for 10 days or azacitidine IV for 7 days. Starting 24 hour after salsalate, patients also receive venetoclax PO continuously until completion of cycle 1. CYCLE 2: Patients receive decitabine IV for 5 days or azacitidine IV for 7 days, salsalate PO BID, and venetoclax PO continuously. Cycles repeat every 28 days in the absence of disease progression or unacceptable	Incidence of adverse events of salicylate + venetoclax + decitabine	3 years	October 2020/ May 2022

					toxicity.			
NCT03217838	Safety, Tolerability, Pharmacokinetics, and Efficacy of AZD2811 Nanoparticles as Monotherapy or in Combination in Acute Myeloid Leukemia Patients.	Phase I/II, Open-Label, non-randomized, parallel assignment	18-130	50 (including R/R and untreated AML)	Part A - Group 1 Arm A: Monotherapy Dose Escalation AZD2811 single agent on Days 1 and 4 of each 28 day cycle. Part A - Group 1 Arm B: Monotherapy Dose Escalation AZD2811 single agent on Days 1, 4, 15, and 18 of each 28 day cycle. Part A - Group 2 Arm A: AZD2811 plus Azacitidine Dose Escalation AZD2811 on Days 1 and 4 plus Azacitidine combination therapy. Escalating doses of AZD2811 Part A - Group 3 Arm A: AZD2811 plus Venetoclax Dose Escalation AZD2811on Days 1 and 4 plus Venetoclax combination therapy. Escalating doses of AZD2811 Part B - Group 1: AZD2811 Monotherapy Dose Expansion Monotherapy dose expansion. Additional patients will be enrolled at the AZD2811 MTD in the dose/schedule that was found most tolerable and/or efficacious. Part B - Group 2: AZD2811 plus Combination Drug Dose Expansion Combination therapy (either azacitidine or venetoclax) dose expansion. Additional patients will be enrolled at the AZD2811 combination MTD based on the dose/schedule from Part A that was found to be most tolerable and/or efficacious. Part A - Group 2 Arm B: AZD2811 plus Azacitidine Dose Escalation AZD2811 on Days 1, 4, 15, and 18 plus azacitidine combination therapy. Escalating doses of AZD2811 Part A - Group 3 Arm B: AZD2811 plus Venetoclax Dose Escalation AZD2811 on Days 1, 4, 15, and 18 plus Venetoclax combination therapy.	Incidence of dose limiting toxicities (DLT) Incidence of adverse events (AEs) Incidence of abnormal laboratory test results Antitumour activity of AZD2811 in patients by assessing total complete remission (CR).	6 months	July 2017/ May 2021

					Escalating doses of AZD2811			
NCT04774393	Decitabine/Cedazuridine and Venetoclax in Combination With Ivosidenib or Enasidenib for the Treatment of Relapsed or Refractory Acute Myeloid Leukemia	Phase 1b/2, open label, non-randomized, parallel assignment	18 and older	84	Arm A (decitabine/cedazuridine, venetoclax, ivosidenib) Patients receive decitabine/cedazuridine PO daily on days 1-5, venetoclax PO daily on days 1-14, and ivosidenib PO daily on days 1-28. Treatment repeats every 28 days for 12 cycles in the absence of disease progression or unacceptable toxicity. Arm B (decitabine/cedazuridine, venetoclax, enasidenib) Patients receive decitabine/cedazuridine PO daily on days 1-5, venetoclax PO daily on days 1-14, and enasidenib PO daily on days 1-28. Treatment repeats every 28 days for 12 cycles in the absence of disease progression or unacceptable toxicity.	Dose limiting toxicity (Phase Ib) Overall response rate (ORR) (Phase II) Incidence of adverse events (Phase II)	3 years	July 2021/ November 2024
NCT03955783	Venetoclax and Selinexor in Treating Patients With Relapsed or Refractory High Risk Hematologic Malignancies	Phase Ib Trial, open label, single group assignment	18 and older	78 (including R/R AML, DLBCL)	Patients receive venetoclax PO QD on days 1-28. Patients with DLBCL receive selinexor PO on days 1, 8, 15, and 22 of each cycle. Venetoclax naïve AML patients receive selinexor on days 8, 15, and 22 of cycle 1, followed by days 1, 8, 15, and 22 of subsequent cycles. Venetoclax refractory AML patients receive selinexor PO on days 1, 8, 15, and 22 of each cycle. Cycles repeat every 28 days in the absence of disease progression or unacceptable toxicity.	Maximum tolerated dose (escalation) Overall response rate (expansion)	2 years	June 2019/ December 2023
NCT04336982	A Safety and Efficacy Study of CC-90009 Combinations in Subjects With Acute Myeloid Leukemia	Exploratory Phase 1/2, open label, non-randomized, parallel assignment	18 and older	66 (including R/R, untreated AML)	CC-90009 in combination with venetoclax and azacitidine CC-90009 will be administered intravenously per dosing schedule in a 28-day cycle. Venetoclax will be administered orally QD. Azacitidine will be administered intravenously or subcutaneously on planned dosing days for each cycle. CC-90009 in combination with gilteritinib CC-90009 will be administered intravenously per dosing schedule in a	Dose Limiting Toxicity (DLT) Maximum Tolerated Dose (MTD) Adverse Events (AEs)	3 years	August 2020/ November 2023

					28-day cycle. Gilteritinib will be administered orally QD.			
NCT03926624	Trial of DFP-10917 vs Non-Intensive or Intensive Reinduction for AML Patients in 2nd/3rd/4th Salvage	Phase III, randomized, controlled, open label	18 and older	450	Experimental<br>DFP-10917 Dose: 6 mg/m²/day administered by continuous infusion for 14 days followed by a 14-day resting period per 28-day treatment cycle. If a patient experiences a significant treatment-related AE, the patient may undergo one dose reduction of DFP-10917 to 4 mg/m²/day x 14 days for subsequent treatment cycles<br>Control<br>Non-Intensive:<br>LoDAC: 20 mg SC BID 10 days<br>Azacitidine: 75 mg/m²/day SC 7 days(or 5+2)<br>Decitabine: CIV 20 mg/m²x5 days<br>Venetoclax + LoDAC/Azacitidine/Decitabine:LoDAC-Venetoclax ramp-up to 600 mgxday. Cytarabine SC 20 mg/m²xday D1-10. Azacitidine or Decitabine-Venetoclax ramp-up to 400 mgxday. Azacitidine IV or SC 75 mg/m² D1-7. Decitabine IV 20 mg/m² on D1-5 or 1-10.<br>Intensive:<br>High DAC: cytarabine 1-2 g/m² up to 5 days, max total dose 10 g/m²<br>FLAG: D1-5: fludarabine 30 mg/m² IV for 30min, D1-5: cytarabine 1-2 g/m² for 4hr daily x 5 & G-CSF 5 mcg/kg or 300 mcg/m² until PMN recovery, with or without idarubicin D1-3 8 mg/m² IV dailyx3 (FLAG-Ida)<br>MEC: D1-6: mitoxantrone 6 mg/m² IV bolus, etoposide 80 mg/m² IV 1hr & cytarabine 1g/m² IV 6hr.<br>CLAG/M or Ida = cladribine 5 mg/m² D1-5, cytarabine 2 g/m² D1-5, G-CSF 300 μg D0-5, mitoxantrone 10 mg/m² D1-3 or Idarubicin 10 mg/m² D1-3.<br>Intermediate DAC: cytarabine 20 mg/m² IV dailyx5	Complete remission (CR) rate<br>Duration of complete remission	3 years	November 2019/ December 2022
NCT04655391	Glasdegib-Based Treatment Combinations for	Pilot/Phase 1b, non-randomized, open label,	18 and older	36	Chapter 1 (glasdegib, decitabine, venetoclax) MOLECULAR DIAGNOSIS	Ability to obtain a molecular diagnosis (Molecular Diagnosis	30 days	February 2021/ December

	the Treatment of Patients With Relapsed Acute Myeloid Leukemia Who Have Undergone Hematopoietic Cell Transplantation	parallel assignment	SEGMENT: Patients receive glasdegib PO QD for at least 14 days until their AML TAC recommendation is made and they are either consented to a treatment arm in the Treatment Segment or go off study.<br>TREATMENT SEGMENT: Patients receive glasdegib PO QD on days 1-28,decitabine IV over 1 hour on days 1-5, and venetoclax PO QD on days 1-14. Treatment repeats every 28 days for up to 12 cycles in the absence of disease progression or unacceptable toxicity.<br>Chapter 2 (glasdegib, gilteritinib)<br>MOLECULAR DIAGNOSIS SEGMENT: Patients receive glasdegib PO QD for at least 14 days until their AML TAC recommendation is made and they are either consented to a treatment arm in the Treatment Segment or go off study.<br>TREATMENT SEGMENT: Patients receive glasdegib PO QD and gilteritinib PO QD on days 1-28. Treatment repeats every 28 days for up to 12 cycles in the absence of disease progression or unacceptable toxicity.<br>Chapter 3 (glasdegib, bosutinib)<br>MOLECULAR DIAGNOSIS SEGMENT: Patients receive glasdegib PO QD for at least 14 days until their AML TAC recommendation is made and they are either consented to a treatment arm in the Treatment Segment or go off study.<br>TREATMENT SEGMENT: Patients receive glasdegib PO QD and bosutinib PO QD on days 1-28. Treatment repeats every 28 days for up to 12 cycles in the absence of disease progression or unacceptable toxicity.<br>Chapter 4 (glasdegib, ivosidenib)<br>MOLECULAR DIAGNOSIS SEGMENT: Patients receive glasdegib PO QD for at least 14 days until their AML TAC	Segment)<br>Ability to make a treatment arm assignment by the Treatment Assignment Committee (TAC) (Molecular Diagnosis Segment)<br>Incidence of adverse events	2022

					recommendation is made and they are either consented to a treatment arm in the Treatment Segment or go off study. TREATMENT SEGMENT: Patients receive glasdegib PO QD and ivosidenib PO QD on days 1-28. Treatment repeats every 28 days for up to 12 cycles in the absence of disease progression or unacceptable toxicity. Chapter 5 (glasdegib, enasidenib) MOLECULAR DIAGNOSIS SEGMENT: Patients receive glasdegib PO QD for at least 14 days until their AML TAC recommendation is made and they are either consented to a treatment arm in the Treatment Segment or go off study. TREATMENT SEGMENT: Patients receive glasdegib PO QD and enasidenib PO QD on days 1-28 .Treatment repeats every 28 days for up to 12 cycles in the absence of disease progression or unacceptable toxicity.			
NCT02115295	Cladribine, Idarubicin, Cytarabine, and Venetoclax in Treating Patients With Acute Myeloid Leukemia, High-Risk Myelodysplastic Syndrome, or Blastic Phase Chronic Myeloid Leukemia	Phase II, open label, Single Group Assignment	18-65	408 (including R/R, untreated AML, CML)	INDUCTION: Patients receive cladribine IV and cytarabine IV over 1-2 hours on days 1-5 and idarubicin IV over 30-60 minutes on days 1-3. Patients with untreated AML and MDS also receive venetoclax PO on days 2-8. AML patients with known FLT3-ITD or FLT3 kinase domain mutations may receive midostaurin PO BID on days 6-19 or gilteritinib PO QD on days 1-14. Treatment repeats every 28 days for up to 2 cycles in the absence of disease progression or unacceptable toxicity. CONSOLIDATION: Patients receive cladribine IV and cytarabine IV over 1-2 hours on days 1-3 and idarubicin IV over 30-60 minutes on days 1-2. Patients with untreated AML and MDS also receive venetoclax PO on days 2-8. AML patients with known FLT3-ITD or FLT3 kinase domain mutations may	Complete response (CR) rate	12 months	May 2014/ May 2022

					receive midostaurin PO BID on days 6-19 or gilteritinib PO QD. Treatment repeats every 28 days for up to 5 cycles in the absence of disease progression or unacceptable toxicity.			
NCT04092179	Study of Enasidenib and Venetoclax in IDH2-Mutated Blood Cancers	Phase Ib/II, open label, sequential assignment	18 and older	48 (including R/R, untreated AML)	Venetoclax and Enadisenib Enasidenib and venetoclax will be taken by mouth (orally), once a day, every day, continuously.<br><br>Every 28-day period will be called a cycle. Participants will start venetoclax alone on Cycle 1 Day 1 and continue the study drug alone until Day 15. On Day 15, participants will take enasidenib and venetoclax together and will continue to take the combination of study drugs until intolerable side effects or disease worsening.	Overall response rate (ORR)<br>Dose Limiting Toxicity<br>Maximum tolerated dose or Recommended Phase 2 Dose<br>Duration of Response	3 years	November 2020/ February 2024
NCT04188405	Decitabine, Venetoclax, and Ponatinib for the Treatment of Philadelphia Chromosome-Positive Acute Myeloid Leukemia or Myeloid Blast Phase Chronic Myelogenous Leukemia	Phase II, open label, Single Group Assignment	18 and older	30 (including R/R, untreated AML)	Treatment (ponatinib, venetoclax, decitabine)	Overall response rate	4.5 years	May 2020/ September 2024
NCT04748848	A Safety, Tolerability and Preliminary Efficacy Study of CC-90011 in Combination With Venetoclax and Azacitidine in R/R Acute Myeloid Leukemia and Treatment-naïve Participants Not Eligible for Intensive Therapy	Phase 1/2, randomized, Open-label, sequential assignment, Multicenter Dose Escalation and Dose Expansion Study	18 and older	132 (including R/R and untreated AML)	CC-90011 in combination with Venetoclax and Azacitidine in Dose Escalation<br>CC-90011 will be given PO on Days 1, 8, and 15 of continuous 4-week (28-day) cycle. The dose escalation is designed to explore three dose levels of CC-90011, for example 20, 40, and 60 mg as determined by Bayesian design.<br>Venetoclax and Azacitidine control arm in dose expansion. The participants will be randomized to the treatment arm or control arm at a 2:1 ratio.<br>CC-90011 in combination with Venetoclax and Azacitidine in Dose Expansion<br>CC-90011 will be given PO on Days 1, 8, and 15 of	Adverse Events (AEs)<br>Recommended Phase 2 dose (RP2D)	2 years	May 2021/ March 2025

					continuous 4-week (28-day) cycle at the recommended phase to dose of CC-90011 confirmed in dose escalation.			
NCT03176277	A Study of ONO-7475 in Patients With Acute Leukemias	Phase I/II, Open Label, Non-Randomized, single group assignment	18 and older	67 (R/R AML and MDS)	ONO-7475 (Part A) Successive dose escalation cohorts to determine MTD/OBD ONO-7475 + venetoclax (Part D) Successive dose escalation of ONO-7475 cohorts + venetoclax	Incidence, nature, and severity of (serious) Adverse Events (Part A) Clinically significant ophthalmology examinations (Part A) Clinically significant electrocardiogram (ECG) (Part A) Complete response (CR)/complete response with partial hematologic recovery (CRh) rate (Part D) Incidence, nature and severity of (serious) Adverse Events (Part D)	12 months	June 2017/ February 2023
NCT04702425	VOB560-MIK665 Combination First in Human Trial in Patients With Hematological Malignancies (Relapsed/Refractory Non-Hodgkin Lymphoma, Relapsed/Refractory Acute Myeloid Leukemia, or Relapsed/Refractory Multiple Myeloma)	Phase Ib, non-randomized, open label, parallel assignment	18 and older	170 (including)	VOB560-MIK665 - Part 1a Part 1a - Patients with relapsed/refractory non-Hodgkin lymphoma and relapsed/refractory multiple myeloma administered VOB560 and MIK665 as an intravenous (IV) infusion. VOB560-MIK665 - Part 1b Part 1b - Patients with relapsed/refractory acute myeloid leukemia administered VOB560 and MIK665 as an intravenous (IV) infusion. VOB560-MIK665 - Part 2a Part 2a - Patients with relapsed/refractory multiple myeloma with at least 10 patients with 1q gain cytogenetic abnormality and 10 patients with high risk R/R MM as defined in (Sonneveld et al 2016) administered VOB560 and MIK665 as an intravenous (IV) infusion. VOB560-MIK665 - Part 2b Part 2b - Patients with relapsed/refractory non-Hodgkin lymphoma with at least 10 patients with double-hit (DH) lymphoma, based on the overall bad prognosis and limited therapeutic options for patients with DH NHL administered VOB560 and MIK665 as an intravenous (IV) infusion. VOB560-MIK665 - Part 2c Part 2c - Patients with	Incidence and severity of AEs and SAEs, including changes in lab values, vital signs, and ECGs Incidence of Dose Limiting Toxicities (DLTs) during the first cycle of treatment with VOB560 and MIK665 in combination Frequency of dose interruptions Frequency of dose reductions Dose intensities	18 months	June 2021/ December 2024

					relapsed/refractory acute myeloid leukemia venetoclax refractory or insensitive with at least 6 patients M5 as proposed by French-American-British (FAB) group, based on the observation that venetoclax resistance in AML M5 can be caused by up-regulation of MCL1 administered VOB560 and MIK665 as an intravenous (IV) infusion. VOB560-MIK665 - Part 2d Part 2d - Patients with relapsed/refractory acute myeloid leukemia venetoclax naive patients administered VOB560 and MIK665 as an intravenous (IV) infusion.			
NCT03826992	Venetoclax Combined With Vyxeos (CPX-351) for Participants With Relapsed or Refractory Acute Leukemia	Phase I, open label, single group assignment	1-39	18 (including AML and ALL)	Venetoclax and Vyxeos combination Venetoclax will be given orally on Days 1-21 per the assigned dose level. A single course consisting of 3 doses of Vyxeos and 21 doses of venetoclax will be administered to participants in this study. Vyxeos will be administered by central venous catheter over 90 minutes on Day 1, 3, and 5. Venetoclax is given daily by mouth per assigned dose level.	Feasibility of combining venetoclax and Vyxeos (dose limiting toxicities) Treatment related toxicities	60 days	December 2018/ January 2023
NCT04477291	A Study of CG-806 in Patients With Relapsed or Refractory Acute Myeloid Leukemia	Phase 1a/b Trial, open label, sequential assignment	18 and older	80	Dose Escalation and Expansion; CG-806 will be given orally in ascending doses in patients with relapsed or refractory AML (escalation cohort), until the maximum tolerated dose or candidate recommended Phase 2 dose is reached. Followed up by up to 50 patients enrolled in the expansion cohort at the recommended dose.	Incidence of treatment-emergent adverse events of CG-806 [Time Frame: At the end of Cycle 1 (each cycle is 28 days)] Establish a CG-806 dose that maintains a biologically active plasma concentration Establish a recommended dose for future development	28 days	October 2020/ June 2023
NCT02665065	Study of Iomab-B vs. Conventional Care in Older Subjects With Active, Relapsed or Refractory Acute Myeloid Leukemia (SIERRA)	Multicenter, Pivotal Phase 3 Study, randomized, open label, parallel assignment	55 and older	150	Iomab-B in conjunction with a Reduced Intensity Conditioning (RIC) regimen containing Fludarabine and low-dose Total Body Irradiation (TBI) prior to allogeneic HCT Conventional Care Defined as Investigator's choice of salvage chemotherapy with any combination of the following agents: Azacitidine (not	Durable Complete Remission (dCR)	5 years	June 2016/ December 2026

					allowed as a single agent), Carboplatin, Cladribine, Clofarabine, Cyclophosphamide, Cytarabine, Daunorubicin, Decitabine (not allowed as a single agent with the exception of patients with documented TP53 mutations who have not previously received 10-day regimens of single agent decitabine), Doxorubicin, Enasidenib, Etoposide, Fludarabine, Gemtuzumab ozogamicin, Idarubicin, Ivosidenib (for subjects with IDH1 mutation), L-Asparaginase, Midostaurin (for FLT3 mutant or FLT3-ITD subjects only, not allowed as single agent), Mitoxantrone, Sorafenib (for FLT3 mutant or FLT3-ITD subjects only, not allowed as single agent), Thioguanine, Topotecan, Venetoclax (in combination with a hypomethylating agent). Chemotherapy agents not listed above may be administered after providing clinical justification and receiving medical monitor approval prior to initiation of treatment.			
NCT03013998	Study of Biomarker-Based Treatment of Acute Myeloid Leukemia	Phase 1b/2, open-label, non-randomized, parallel assignment	18 and older	2,000 (including R/R, untreated AML, MDS)	Samalizumab<br>BI 836858<br>AG-221<br>Entospletinib<br>Decitabine<br>Entospletinib<br>Daunorubicin<br>Cytarabine<br>Pevonedistat<br>AG-120<br>Azacitidine<br>Gilteritinib<br>Decitabine<br>AZD5153<br>Venetoclax<br>TP-0903<br>Decitabine	Proportion of patients for whom molecular, immunophenotypic, and/or biochemical studies are completed in < 7 calendar days for assignment of treatment<br>Proportion of patients assigned to a novel therapeutic treatment group in 1 of several sub-studies in this Master Protocol, based on the result of the molecular, immunophenotypic, and/or biochemical studies<br>Clinical response rate	5 years	November 2016/ December 2021
NCT03236857	A Study of the Safety and Pharmacokinetics of Venetoclax in Pediatric and Young Adult Patients With Relapsed or	Phase 1 Study, open label, single group assignment	Up to 25	165 (including ALL, AML, NHL, neuroblastoma)	Venetoclax with or without chemotherapy<br>Venetoclax administered orally once daily (QD) with various doses and dosing regimens with or without chemotherapy at the discretion of the investigator.	AUC0-24 post-dose of venetoclax [Time Frame: Up to approximately 2 weeks]<br>Area under the plasma concentration-time curve from 0 to 24 hours (AUC24) post-dose of	9 months	November 2017/ July 2022

	Refractory Malignancies				Allowed chemotherapy regimens as outlined in the study protocol.	venetoclax<br>Recommended Phase 2 dose (RPTD) of venetoclax<br>Number of Participants with Dose limiting toxicities (DLT) of Venetoclax Monotherapy<br>Tmax of venetoclax<br>Cmax of venetoclax		
NCT03471260	Ivosidenib and Venetoclax With or Without Azacitidine in Treating Participants With IDH1 Mutated Hematologic Malignancies	Phase Ib/II, open label, Single Group Assignment	18 and older	48 (including R/R and untreated)	Treatment (venetoclax, ivosidenib, azacitidine)<br>Participants receive venetoclax PO daily on days 1-14. Participants also receive ivosidenib PO daily on days 15-28 of cycle 1 and days 1-28 of subsequent cycles. Participants may also receive azacitidine IV over 30-60 minutes or SC on days 1-7. Cycles repeat every 28 days in the absence of disease progression or unacceptable toxicity.	Overall response rate (ORR)<br>Incidence of adverse events<br>Dose-limiting toxicity	3 years	March 2018/ September 2021
NCT04424147	Efficacy and Safety of HVA Regimens as Salvage Treatment in rrAML	Phase 2, open label, Single Group Assignment	18-80	40	HVA Regimens as Salvage Treatment<br>Venetoclax with a dose of 400mg per day for 14 days, combined with azacitidine (AZA) with a dose of 75mg/m2 per day for 7 days, and HHT 1mg/m2 per day for 7 days	Complete response	1 years	May 2020/ May 2023
NCT04029688	A Study Evaluating the Safety, Tolerability, Pharmacokinetics, and Preliminary Activity of Idasanutlin in Combination With Either Chemotherapy or Venetoclax in the Treatment of Pediatric and Young Adult Participants With Relapsed/Refractory Acute Leukemias or Solid Tumors	Phase I/II, Multicenter, randomized, Open-Label, sequential assignment	Up to 30	220 (R/R acute leukemia or solid tumors)	Idasanutlin will be administered as an oral medication once daily on Days 1-5 of a 28-day cycle.<br>Venetoclax will be administered orally at the adult dose equivalent (adjusted by body weight) of 400 milligrams (mg) in participants with neuroblastoma and the adult dose equivalent of 600 mg in participants with leukemia.<br>Cyclophosphamide will be administered once daily on Days 1-5 of each 28-day cycle at 250 milligrams per meter squared of body surface area (mg/m^2) as an intravenous (IV) infusion.<br>Topotecan will be administered once daily on Days 1-5 of each 28-day cycle at 0.75 mg/m^2 as an IV infusion.<br>Fludarabine will be administered once daily on	Number and Severity of Participants with at Least One Adverse Event<br>Number of Participants with Dose-Limiting Toxicities (DLTs)<br>Parts 2 and 3: Percentage of Participants with TP53 Wild-Type (WT) Neuroblastoma Achieving an Objective Response<br>Parts 2 and 3: Complete Remission Rate Within 2 Cycles of Study Treatment in Participants with TP53 WT Leukemia<br>Parts 2 and 3: Percentage of Participants with TP53 WT ALL who are Minimal Residual Disease (MRD)-Negative Within	1 year	January 2020/ May 2024

	Days 1-5 of each 28-day treatment cycle at 30 mg/m^2 as an IV infusion. Cytarabine will be administered once daily on Days 1-5 of each 28-day treatment cycle at 2000 mg/m^2 as an IV infusion.	2 Cycles of Study Treatment	
